# Genome-Wide Analysis of AGC Kinases Reveals that MoFpk1 Is Required for Development, Lipid Metabolism, and Autophagy in Hyperosmotic Stress of the Rice Blast Fungus Magnaporthe oryzae

**DOI:** 10.1128/mbio.02279-22

**Published:** 2022-10-19

**Authors:** Ming-Hua Wu, Qin Yu, Tian-Yi Tao, Li-Xiao Sun, Hui Qian, Xue-Ming Zhu, Lin Li, Shuang Liang, Jian-Ping Lu, Fu-Cheng Lin, Xiao-Hong Liu

**Affiliations:** a State Key Laboratory for Managing Biotic and Chemical Treats to the Quality and Safety of Agro-products, Institute of Biotechnology, Zhejiang University, Hangzhou, Zhejiang Province, China; b State Key Laboratory for Managing Biotic and Chemical Treats to the Quality and Safety of Agro-products, Institute of Plant Protection and Microbiology, Zhejiang Academy of Agricultural Sciences, Hangzhou, Zhejiang Province, China; c College of Life Sciences, Zhejiang University, Hangzhou, Zhejiang Province, China; Universidade de Sao Paulo

**Keywords:** *Magnaporthe oryzae*, AGC kinases, MoFpk1, lipid metabolism, autophagy

## Abstract

During eukaryotic evolution, the TOR-AGC kinase signaling module is involved in the coordinated regulation of cell growth and survival. However, the AGC kinases in plant-pathogenic fungi remain poorly understood. In this study, we have identified 20 members of the AGC family of protein kinases. Evolutionary and biological studies have revealed that AGC kinases are highly conserved and involved in the growth (8 genes), conidiation (13 genes), conidial germination (9 genes), appressorium formation (9 genes), and pathogenicity (5 genes) of Magnaporthe oryzae, in which a subfamily protein of the AGC kinases, MoFpk1, the activator of flippase, specifically exhibited diverse roles. Two kinase sites were screened and found to be critical for MoFpk1: 230K and 326D. Moreover, MoFpk1 is involved in cell wall integrity through the negative regulation of MoMps1 phosphorylation. The deletion of MoFpk1 resulted in defective phosphatidylacetamide (PE) and phosphatidylserine (PS) turnover and a series of lipid metabolism disorders. Under hyperosmotic stress, since the Δ*Mofpk1* mutant is unable to maintain membrane asymmetry, MoYpk1 phosphorylation and MoTor activity were downregulated, thus enhancing autophagy. Our results provide insights into the evolutionary and biological relationships of AGC kinases and new insight into plasma membrane (PM) homeostasis, i.e., responses to membrane stress and autophagy through lipid asymmetry maintenance.

## INTRODUCTION

Magnaporthe oryzae, the causal agent of rice blast, serves as a model for understanding microbe-plant interactions (1). It is one of the most destructive pathogens of rice and grasses, causing huge economic losses annually (1). M. oryzae, a filamentous ascomycete fungus, has a complex infection cycle, including conidiogenesis, germination, appressorium formation, and invasion.

Autophagy is a conserved pathway that is induced by external environmental changes. It can be triggered and controlled by many metabolic processes; one of them is the target of rapamycin (TOR). TOR is a class of evolutionarily conserved serine/threonine (Ser/Thr) protein kinases (2); it controls cell growth by activating catabolic processes such as autophagy (3, 4). There are two TOR complexes in Saccharomyces cerevisiae, TORC1 and TORC2. TORC1 is sensitive to rapamycin and is involved in cell growth metabolism; TORC2 participates in the hyperosmotic stress response (5, 6). Emerging findings indicate multiple modes of cross talk between AGC kinases and TOR (7). One of the first bona fide cellular targets of TOR was the mammalian p70S6K kinase (7), a member of the AGC kinases (protein kinase A [PKA]/protein kinase G [PKG]/protein kinase C [PKC]), which defined a group of serine/threonine protein kinases that share sequence similarity in their catalytic kinase domains with cAMP-dependent PKA, cGMP-dependent PKG, and phospholipid-dependent PKC (8). The AGC kinase family is one of seven kinase families conserved in all eukaryotic genomes and is widely found in mammals (7, 9), plants (10), and fungi (7). In S. cerevisiae, there are about 22 AGC kinases (11, 12), including *PKC1*, *YPK1/2*, *SCH9*, *TPK1/2/3*, *CBK1*, *RIM15*, *DBF2/DBF20*, *PKH1/2/3*, *IPL1*, *BUB1*, *KIN82* (*FPK1*), *YNR047W*, *YBR028C*, and *YKL171W*. Most AGC kinases undergo phosphorylation activation and one or more phosphorylation sites are rapamycin sensitive; therefore, AGC kinases were likely regulated by TOR directly (7). At present, many evidences proved that AGC family participates in growth and stress response under TOR complex regulation, for instance, TORC1 mediates the participation of Sch9 in cell growth (13); TORC2 regulates the actin cytoskeleton through Pkc1 (14); external stress-activated TOR activity then stimulates or diminishes Ypk1 phosphorylation (15–17), etc. In M. oryzae, five AGC kinases (MoCpkA, MoCpk2, MoRim15, MoSch9, and MoTos3) have been reported to be involved in hyphal growth, conidiation, appressorium formation, and pathogenicity (18–20). However, systemic knowledge of AGC kinases in M. oryzae remains unclear.

The eukaryotic plasma membrane (PM) with an asymmetric distribution of phospholipids is composed of specific proteins and lipids with high degrees of composition and spatial organization (21, 22). The continuous dynamic remodeling of lipids in the two leaflets of the PM is vital to the response to environmental stresses such as sphingolipid (SP) depletion (21–23). In S. cerevisiae, the asymmetric distribution of phospholipids was maintained by five type 4 P-type ATPases (flippases), Dnf1, Dnf2, Dnf3, Drs2, and Neo1, through transferring phosphatidylacetamide (PE) and phosphatidylserine (PS) from extracellular to intracellular locations (22, 24). The AGC kinase Fpk1 and its homologue Fpk2 act as upstream regulatory proteins of Lem3-Dnf1 and Lem3-Dnf2, which are putative flippase complexes that localize to the plasma membrane (24). However, how the phospholipid flippase rate affects the lipid distribution in the cell membrane and, in turn, other metabolic processes in M. oryzae remains unknown.

Here, we performed a genome-wide analysis of the AGC kinase family in M. oryzae. AGC kinases are involved in the growth, conidiation, germination, appressorium formation, and pathogenicity of M. oryzae. Particularly, a ribosomal S6 kinase (RSK) subfamily member, MoFpk1, contributed to the pleiotropic roles. In this study, we propose a novel mechanism for PM homeostasis, i.e., adaptation to stress through the asymmetric distribution of phospholipids, and also the internal linkage between lipid metabolism and autophagy, which provides an invaluable foundation and resource for deepening the understanding of the complex regulatory networks of fungal development and pathogenicity mechanisms.

## RESULTS

### Identification and phylogenetic analysis of AGC kinases in M. oryzae.

To extensively identify M. oryzae AGC kinases, Kionmer v1.0 (http://www.compbio.dundee.ac.uk/kinomer/index.html) (25) was used to perform a genome-wide search to identify AGC kinases in M. oryzae using the 22 S. cerevisiae AGC kinase proteins sequences as a query (12). In most eukaryotic protein kinase (ePK) families, protein kinases usually have a similar quantity of number (12). Similar to S. cerevisiae and Schizosaccharomyces pombe (7), 20 AGC kinases in M. oryzae were identified (Table 1), which suggested that AGC kinases are conserved in fungi.

**TABLE 1:** Twenty AGC kinases in M. oryzae^*a*^

Gene locus	Subgroup	NCBI protein accession no.	Gene	% homology with S. cerevisiae	Positions	Amino acid sequence length	Mol wt	Theoretical pI	Growth	Conidiation	Germination	Appressorium formation	Virulence
*MGG_00345*	NDR	XP_003718738.1	*RIM15*	40.54	65–135, 735–1169, 1170–1288, 1535–1649	1,952	212,096	5.82	Reduced	—	—	Normal	Reduced
*MGG_02757*	NDR	XP_003720996.1	*DBF20*	56.34	266–569, 570–649	670	76,015.93	8.1	Reduced	Reduced	Normal	Reduced	Normal
*MGG_04475*	NDR	XP_003710985.1	*KSP1*	31.72	350–712, 756–805	1,011	112,275.6	9.12	Normal	Reduced	Reduced	Reduced	Normal
*MGG_05376*	NDR	XP_003710223.1	*CBK1*	56.49	267–571, 572–652	652	73,867.48	8.76	—	—	—	—	—
*MGG_06514*	NDR	XP_003717039.1	*SCH9*	36.70	20–275	567	62,933.6	8.12	Normal	Reduced	Reduced	Reduced	Normal
*MGG_09519*	NDR	XP_003712223.1	*CBK1*	52.02	98–409, 410–488	488	56,167.85	6.66	Reduced	Increased	Reduced	Normal	Normal
*MGG_00479*	Other	XP_003718583.1	*LPL1*	50.75	124–377	397	45,094.89	9.37	—	—	—	—	—
*MGG_02051*	Other	XP_003708807.1	*CDC5*	30.43	305–628	644	70,588.7	6.73	Normal	Reduced	Reduced	Reduced	Normal
*MGG_06421*	Other	XP_003717147.1	*TOS3*	30.49	106–401	651	72,365.43	5.36	Normal	Increased	Normal	Normal	Normal
*MGG_12408*	Other	XP_003715581.1	*LPL1*	43.82	123–376	400	45,566.5	9.87	Reduced	Increased	Normal	Normal	Normal
*MGG_14329*	Other	XP_003710704.1	*BUB1*	29.72	900–1252, 63–225	1,252	139,369.5	4.92	—	—	—	—	—
*MGG_15988*	Other	XP_003708741.1	*IRE1*	46.98	849–1147, 1150–1283	1,286	143,352.2	6.03	—	—	—	—	—
*MGG_02832*	PKA	XP_003720907.1	*CPK2*	40–53	75–350, 351–408	408	45,852.69	5.97	Normal	Normal	Normal	Normal	Normal
*MGG_06368*	PKA	XP_003717206.1	*CPKA*/*TPK2*	71.47	228–483, 484–539	539	60,759.82	7.79	Reduced	Reduced	Reduced	Reduced	Reduced
*MGG_06599*	AKT	XP_003716929.1	*YPK1*	46.28	297–558, 559–630	647	71,951.44	8.02	—	—	—	—	—
*MGG_14773*	AKT	XP_003714158.1	*SCH9*	62.22	296–465, 498–759, 760–845	903	99,144.32	5.94	Reduced	Reduced	Reduced	Reduced	Reduced
*MGG_08689*	PKC	XP_003719292.1	*PKC*	59.53	1–65, 157–234, 240–358, 479–527, 547–597, 857–1116, 1117–1182	1,182	131,966.8	7.32	—	—	—	—	—
*MGG_01795*	PKD1	XP_003714803.1	*PKH1*	48.48	248–521	838	91,939.99	9.04	—	—	—	—	—
*MGG_01260*	RSK	XP_003714172.1	*YPK3*	40.00	225–486, 487–591	598	65,550.68	8.07	Reduced	Reduced	Normal	Normal	Normal
*MGG_07012*	RSK	XP_003709770.1	*FPK1*	55.76	201–484	547	60,489.58	9.14	Reduced	Reduced	Reduced	Reduced	Reduced

a “—” indicates no data due to the unsuccessful knockout of the gene.

**FIG 1:**
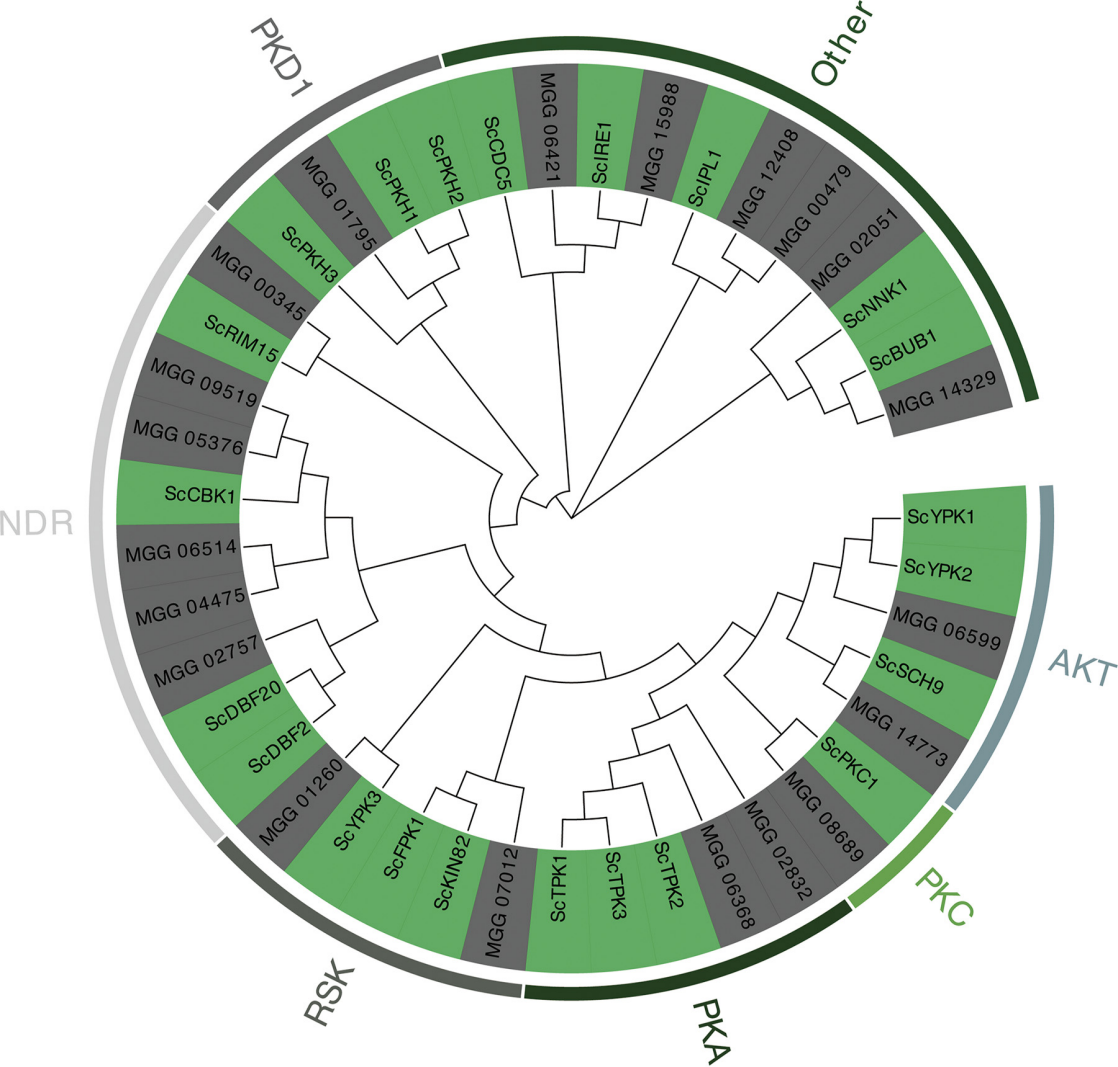
Phylogenetic analysis and classification of M. oryzae and S. cerevisiae AGC kinases. The AGC kinase domains were used to establish the phylogenetic tree using the maximum likelihood method with 1,000 bootstrap replicates, the Poisson correction model, and partial deletion. The classification follows the classification of S. cerevisiae AGC kinases. Labeled lines outside the tree represent clade names of the subfamilies of AGC kinases.

The AGC kinases share high homology in their kinase domains (7). Thus, the amino acid sequences of the kinase domains were used to construct a phylogenetic tree using MEGA11 (26) (Fig. 1). According to the standards of classification for S. cerevisiae (11), M. oryzae AGC kinases were classified into 7 subfamilies, 2 belonged to the PKA (protein kinase A) subfamily, 1 belonged to PKC (protein kinase C) subfamily, 2 belonged to AKT (kinase from the AKT8 retrovirus) subfamily, 2 belonged to RSK (ribosomal S6 kinase) subfamily, 6 belonged to NDR (nuclear Dbf2 related) subfamily, and 1 belonged to PKD1 (3-phosphoinositide-dependent protein kinase 1) subfamily, and 6 genes were classified into the “other” group, which are newly added to the AGC family (11). The new functionalization after the domain duplication of some AGC kinases led to the misclassification of the family (12); thus, we further determined the evolutionary role of AGC kinases.

### Gene structure analysis, conserved motifs, and chromosomal locations of AGC kinases in M. oryzae.

**FIG 2:**
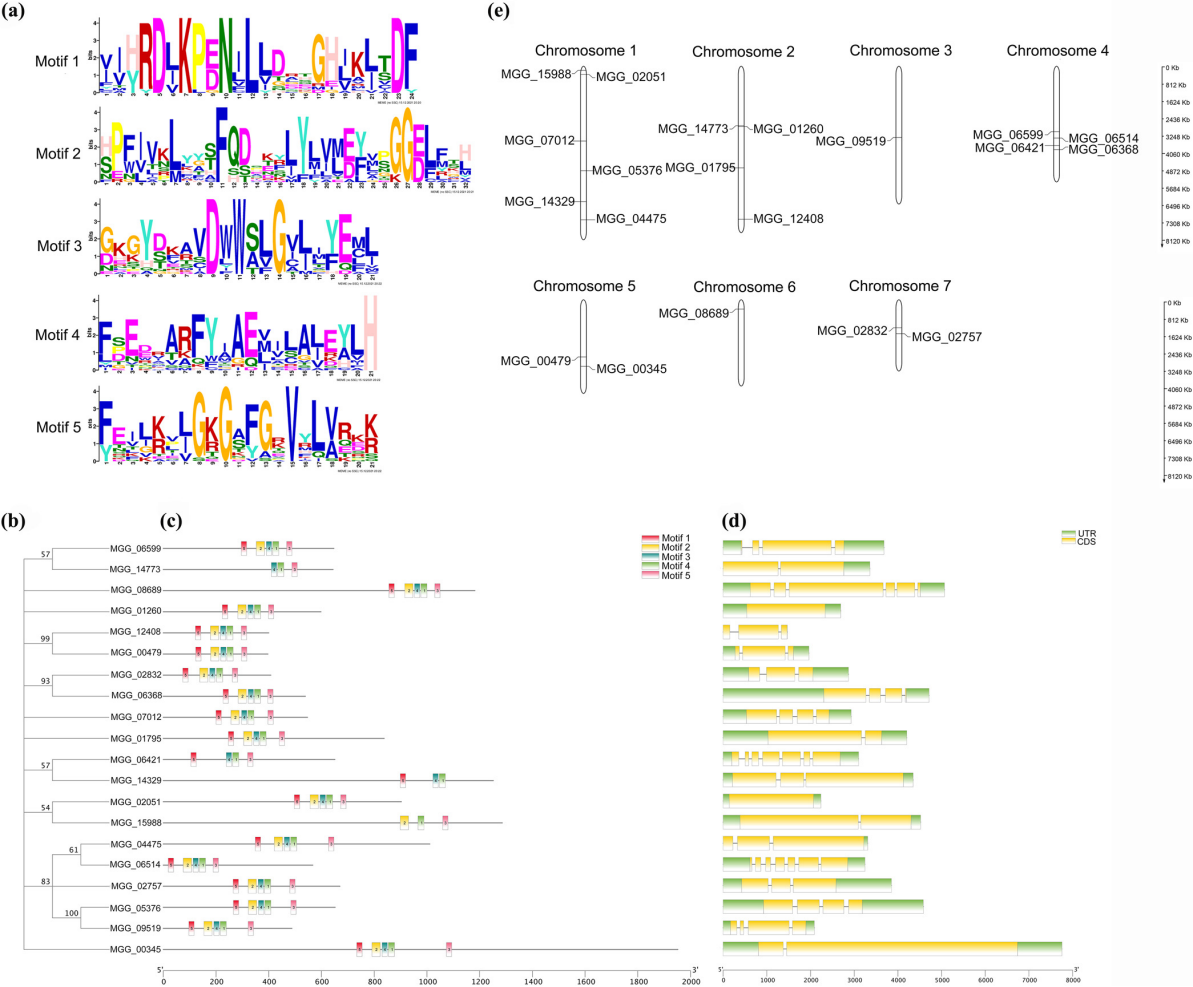
AGC kinases are conserved in M. oryzae. (a) The predicted conserved motifs of 20 AGC kinase catalytic domains were obtained using the MEME (Multiple Em for Motif Induction) tool. (b) The phylogenetic tree was constructed based on the amino acid sequences of AGC kinase catalytic domains using MEGA11. (c) The distribution of the locations of the 5 motifs in 20 AGC kinase catalytic domains was predicted by TBtools. Motifs 1 to 5 are displayed as different-colored boxes. (d) The exon/intron structures of AGC kinases. The arrangement of AGC kinase introns/exons was mapped using TBtools. UTR, untranslated region; CDS, coding DNA sequence. (e) The physical positions of 20 AGC kinases. The Ensembl project was used to depict the chromosomal locations of the 20 AGC kinases.

The members of the AGC protein kinase family share high homology in their kinase domains. A total of 5 different motifs, named motifs 1 to 5, predicted by the MEME tool are shown according to their phylogenetic tree (Fig. 2a to c). Sixteen (80%) AGC enzymes showed similar motif compositions, except for *MGG_14773*, *MGG_06421*, *MGG_14329*, and *MGG_15988*, in which one or two motifs were missing. Interestingly, these genes were divided into the “other” group in the phylogenetic tree (Fig. 1), indicating that the conserved domains of these AGC kinases may be lost or evolved during evolution.

In the process of evolution, the diversification of exons/introns and the precise gain/loss of introns play vital roles in the evolution of certain gene families (27, 28). Thus, the organization of AGC kinase introns/exons was mapped using TBtools (29). AGC kinases showed a diverse number of introns, ranging from 1 to 7 (Fig. 2d). The duplication of AGC kinases plays a prominent role in increasing TOR complexity (30). Next, the physical positions of AGC kinases were mapped (31) (Fig. 2e). All of the AGC kinases were unevenly distributed among the 7 chromosomes of M. oryzae. Many AGC kinases were located on chromosome 1 (6; 30%), chromosome 2 (4; 20%), and chromosome 4 (4; 20%), and only 1 (5%) AGC kinase each was located on chromosomes 3 and 6. In general, we inferred that the conservation of AGC families might originate from genetic conservation.

### AGC kinases are involved in the fungal growth, asexual development, and pathogenicity of M.oryzae.

**FIG 3:**
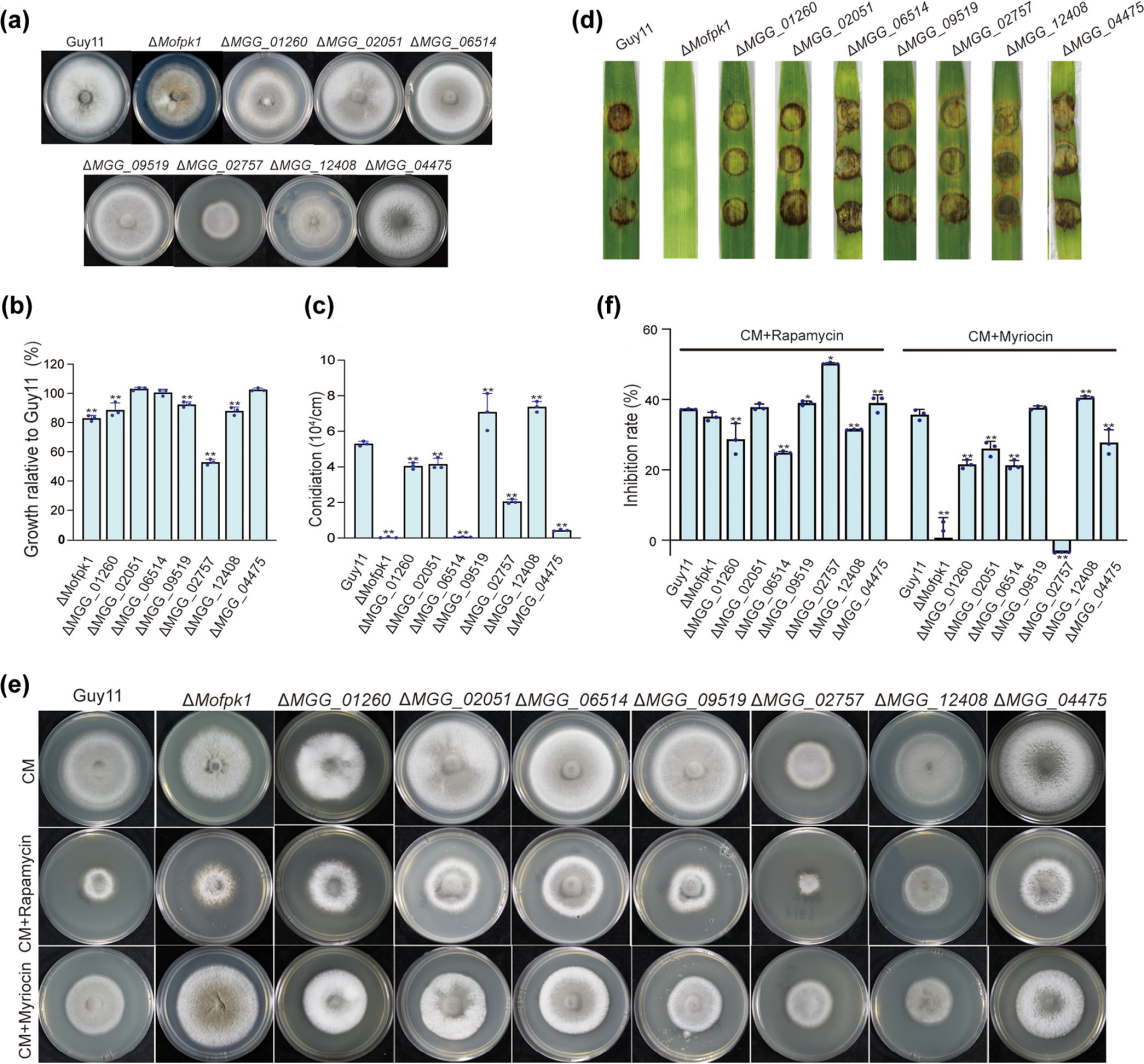
Biological functions of AGC kinases in M.oryzae. (a) Colony morphologies of the AGC kinase mutants cultured on CM plates for 8 days at 25°C. (b) Relative growth rates of AGC kinase mutants (relative growth rate = colony diameter of the mutants/colony diameter of the wild type). The experiment was repeated three times. Data were analyzed by analysis of variance (ANOVA) using SPSS, followed by a Duncan *post hoc* least significant difference (LSD) test for comparison (*, *P* ≤ 0.05; **, *P* ≤ 0.01). (c) Conidiation of the AGC kinase mutants. The colony diameter was measured, and conidia were collected. The conidia were counted under a microscope (conidial production = number of conidia/colony diameter [10^4^ conidia/cm^2^]). The experiment was repeated three times. Data were analyzed as described above for panel c. (d) Pathogenicity of AGC kinase mutants on barley leaves. Disease symptoms on cut barley leaves are shown. The barley leaves were inoculated with mycelial plugs from the strains. Typical leaves were photographed at 96 h postinoculation (hpi). (e) Analysis of the sensitivity of M. oryzae AGC kinase mutants to rapamycin and myriocin. Guy11 and AGC kinase mutants were cultured on CM, CM plus rapamycin (100 ng/mL), and CM plus myriocin (1 μM) for 8 days at 25°C in the dark. The morphology of the colonies was observed and photographed. (f) Growth inhibition rates of the strains supplemented with rapamycin and myriocin. Data were analyzed as described above for panel c.

Next, a gene knockout mutant library of AGC kinases was generated as previously reported (32, 33). Except for 7 genes that could not be successfully knocked out (*MGG_00479*, *MGG_01795*, *MGG_08689*, *MGG_15988*, *MGG_06599*, *MGG_14329*, and *MGG_05376*) and 5 genes that were studied previously (*MGG_00345* [18], *MGG_02832* [20], *MGG_06368* [34], *MGG_06421* [35], and *MGG_14773* [36]), 8 genes were successfully knocked out: *MoFPK1* (*MGG_07012*), *MGG_01260*, *MGG_02051*, *MGG_06514*, *MGG_09519*, *MGG_02757*, *MGG_12408*, and *MGG_04475* (see Fig. S1a and b in the supplemental material). The deletions of *MoFPK1*, *MGG_01260*, *MGG_09519*, *MGG_02757*, and *MGG_12408* showed reduced growth. However, other mutants showed similarities to Guy11 (Fig. 3a and b). Regarding the conidiation ability, 6 mutants produced less or even no conidia, especially the Δ*Mofpk1*, Δ*MGG_06514*, and Δ*MGG_04475* mutants. Differently, the Δ*MGG_12408* and Δ*MGG_09519* mutants produced more conidia (Fig. 3c). Next, pathogenicity assays were performed. Except for the Δ*Mofpk1* mutant, which showed no lesions, the Δ*MGG_01260*, Δ*MGG_02051*, Δ*MGG_06514*, Δ*MGG_09519*, Δ*MGG_02757*, Δ*MGG_12408*, and Δ*MGG_04475* mutants showed lesions similar to those of the wild type (WT) (Fig. 3d). Germination and appressorium formation also exhibited diverse delays in AGC gene deletion mutants (Fig. S1d and e). Furthermore, reintroducing their respective native copies could restore the defect, further confirming that these AGC kinases play key roles in M. oryzae (data not shown).

10.1128/mbio.02279-22.1 FIG S1 Biological function of AGC kinases. (a) Schematic diagram of the knockout principle. (b) Electropherogram of knockout mutants. According to the criteria, the target gene (~500 bp) could not be detected, while a unique recombinant DNA fragment (~2,000 bp) and β-tubulin (~1,000 bp) could be detected in the null mutant by double PCR. (c) Phenotypic summary of 13 AGC kinase gene-deleted mutants of M. oryzae. The phenotypes investigated included mycelial growth, asexual development (conidiation), conidial germination, appressorium formation, and virulence (including conidial and hyphal virulence). (d) Rates of germination of AGC kinases. The experiment was repeated three times. Data were analyzed by ANOVA using SPSS, followed by a Duncan *post hoc* LSD test for comparison (*, *P* ≤ 0.05; **, *P* ≤ 0.01). (e) Rate of appressorium formation of AGC kinases. The experiment was repeated three times. Data were analyzed by ANOVA using SPSS, followed by a Duncan *post hoc* LSD test for comparison (*, *P* ≤ 0.05; **, *P* ≤ 0.01). Download FIG S1, TIF file, 0.7 MB. Copyright © 2022 Wu et al. 2022 Wu et al. https://creativecommons.org/licenses/by/4.0/ This content is distributed under the terms of the Creative Commons Attribution 4.0 International license.

Emerging findings indicated that TOR balances growth and survival signals by regulating AGC kinases (7, 37). We therefore used rapamycin, an inhibitor of TOR, and myriocin, an inhibitor of sphingolipid synthesis, to test the sensitivity of the strains. As shown in Fig. 3e and f, the Δ*MGG_06514*, Δ*MGG_01260*, and Δ*MGG_12408* mutants exhibited resistance to rapamycin; the Δ*MGG_09519*, Δ*MGG_02757*, and Δ*MGG_04475* mutants showed sensitivity to rapamycin; and the Δ*Mofpk1* and Δ*MGG_02051* mutants appeared to have no effect. The Δ*MGG_12408* mutant showed higher sensitivity to myriocin; the Δ*MGG_09519* mutant appeared to have no effect; and the Δ*MGG_02051*, Δ*MGG_06514*, Δ*Mofpk1*, Δ*MGG_02757*, Δ*MGG_04457*, and Δ*MGG_01260* mutants showed significantly lower rates of inhibition. In general, we speculated that AGC kinases play different roles in TOR signaling and sphingolipid synthesis.

### Deletion of *MoFPK1* leads to pleiotropic defects in M. oryzae.

Of these AGC kinases, *MGG_07012*, named MoFpk1, specifically exhibited diverse deficiencies in M. oryzae. MoFpk1 shared a highly conserved domain at the C terminus with S. cerevisiae, Schizosaccharomyces pombe, Neurospora crassa, and Fusarium proliferatum Fpk1 (Fig. S2a). Furthermore, MoFpk1 was evenly distributed in the cytoplasm of hyphae, conidia, and appressoria (Fig. S2b and c), similarly to ScFpk1 (24).

10.1128/mbio.02279-22.2 FIG S2 Biological function of MoFpk1. (a) Multiple-sequence alignment of MoFpk1 proteins using NCBI Protein BLAST analysis. (b) Subcellular localization of MoFpk1 in the mycelium. The mycelium was harvested from culture in liquid CM for 2 days and observed under a fluorescence microscope. MoFpk1 was located in the cytoplasm. Bar = 10 μm. (c) Same as panel b but for the subcellular localization of MoFpk1 in conidia and the appressorium. Bar = 10 μm. (d) Sensitivity of the M. oryzae strains to osmotic stress. Shown are the colony morphologies of the Guy11, Δ*Mofpk1*, MoFpk1C, and MoFpk1^K230R^,MoFpk1^D326A^ strains grown on medium supplemented with different osmotic stress agents, 0.5 M NaCl or 0.5 M KCl, for 8 days at 25°C. The rates of growth inhibition of the strains on medium supplemented with NaCl and KCl are shown. (e) Relative growth rates of strains in response to osmotic stress. The experiment was repeated three times independently. Data were analyzed by ANOVA using SPSS, followed by a Duncan *post hoc* LSD test for comparison (*, *P* ≤ 0.05; **, *P* ≤ 0.01). (f) Carbon source utilization by the Guy11, Δ*Mofpk1*, and MoFPK1C strains. The strains were inoculated onto plates supplemented with glucose, sucrose, xylose, maltose, olive, Tween 80, or 50 mM NaAc as the sole carbon source. (g) Relative growth rates with carbon source utilization of the Guy11, Δ*Mofpk1*, and MoFPK1C strains. Download FIG S2, JPG file, 1.4 MB. Copyright © 2022 Wu et al. 2022 Wu et al. https://creativecommons.org/licenses/by/4.0/ This content is distributed under the terms of the Creative Commons Attribution 4.0 International license.

**FIG 4:**
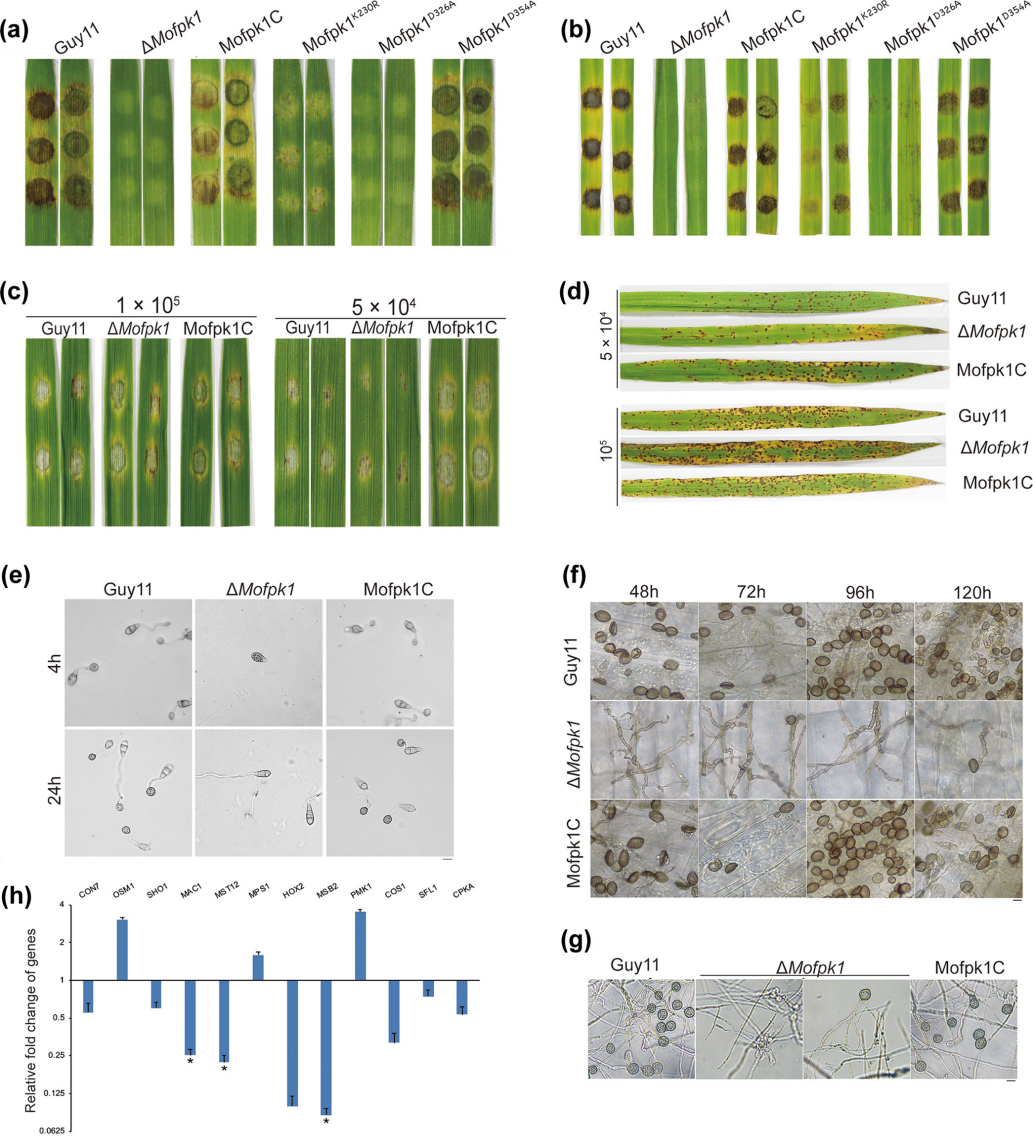
MoFpk1 is involved in the conidiation, appressorium formation, and pathogenicity of M. oryzae. (a) Pathogenicity of the Δ*Mofpk1* mutant. Disease symptoms on cut leaves of barley inoculated with mycelial plugs for 4 days are shown. (b) Disease symptoms on cut leaves of rice inoculated with mycelial plugs for 4 days. (c) Disease symptoms of the Δ*Mofpk1* conidial suspension on cut leaves of barley for 4 days. The cut leaves of barley were inoculated with conidial suspensions of 1 × 10^5^ or 5 × 10^4^ conidia/mL. (d) Disease symptoms on 14-day-old rice seedlings inoculated with conidial suspensions. The leaves of rice were sprayed with conidial suspensions of 5 × 10^4^ conidia/mL or 1 × 10^5^ conidia/mL, placed in the dark for 48 h, and then transferred to 16 h of light/8 h of darkness at 25°C. Typical leaves were photographed after 6 days. (e) Appressorium formation. The conidia were inoculated onto a plastic hydrophobic membrane to induce appressorium formation (bar = 10 μm) and photographed at 4 hpi and 24 hpi. (f) Development of appressorium-like structures on barley leaves (bar = 10 μm). (g) Development of appressorium-like structures on a plastic hydrophobic membrane covered with a mycelial suspension (bar = 10 μm). (h) Analysis of the expression levels of appressorium and appressorium-like structure formation-related genes in the Δ*Mofpk1* mutant. Expression data were normalized using the actin and 40S genes. Error bars represent the standard deviations. Data were analyzed by ANOVA using SPSS, followed by a Duncan *post hoc* LSD test for comparison (*, *P* ≤ 0.05; **, *P* ≤ 0.01). MoFpk1C, MoFpk1 complementary strain.

Pathogenicity assays were then conducted. The medium plug of the Δ*Mofpk1* mutant caused hardly any lesions in both barley and rice (Fig. 4a and b). However, the Δ*Mofpk1* mutant showed pathogenicity similar to that of Guy11 in conidial pathogenicity assays (Fig. 4c) and spray assays (Fig. 4d). Furthermore, only 18.13% of conidia of the Δ*Mofpk1* mutant on an artificial hydrophobic surface developed germ tubes, whereas 92.30% of Guy11 conidia developed germ tubes at 4 h postinoculation (hpi). The rate of appressorium formation of the Δ*Mofpk1* mutant was 7.8%, whereas that of Guy11 was 90.5% (Fig. 4e). Besides, the Δ*Mofpk1* mutant showed severe defects in the formation and infection of appressorium-like structures (ALSs) (Fig. 4f and g). Next, the expression levels of genes associated with the appressorium and ALS were further analyzed by quantitative real-time PCR (qRT-PCR). *CON7*, *SHO1*, *MAC1*, *MST12*, *HOX2*, *MSB2*, *COS1*, *SFL1*, and *CPKA* were downregulated in the Δ*Mofpk1* mutant, and *OSM1*, *MPS1*, and *PMK1* were upregulated, compared to the levels in Guy11 (Fig. 4h). Additionally, the Δ*Mofpk1* mutant showed slower lipid droplet and glycogen degradation (Fig. S3) and also showed carbon source utilization defects (Fig. S2f and g). Above all, the deletion of *MoFPK1* led to severe damage to M. oryzae.

10.1128/mbio.02279-22.3 FIG S3 MoFpk1 is involved in the degradation of glycogen and lipid droplets in M. oryzae. (a) Mobilization and degradation of conidia and appressorium lipid droplets. The Δ*Mofpk1*, MoFpk1C, and Guy11 strains were stained with Bodipy and observed under a fluorescence microscope. Bar = 10 μm. (b) Lipid-containing conidia in the Δ*Mofpk1*, MoFpk1C, and Guy11 strains. Data were analyzed by ANOVA using SPSS, followed by a Duncan *post hoc* LSD test for comparison (*, *P* ≤ 0.05; **, *P* ≤ 0.01). (c) A KI/-I_2_ solution was used to stain the glycogen deposits in the conidia of the Δ*Mofpk1* mutant, Guy11, and MoFpk1C strains. Bar = 10 μm. (d) Glycogen-containing conidia in the Δ*Mofpk1* mutant, Guy11, and MoFpk1C strains. Data were analyzed by ANOVA using SPSS, followed by a Duncan *post hoc* LSD test for comparison (*, *P* ≤ 0.05; **, *P* ≤ 0.01). (e) Rates of glycogen-containing appressoria in the Δ*Mofpk1* mutant, Guy11, and MoFpk1C strains. Data were analyzed by ANOVA using SPSS, followed by a Duncan *post hoc* LSD test for comparison (*, *P* ≤ 0.05; **, *P* ≤ 0.01). Download FIG S3, JPG file, 1.2 MB. Copyright © 2022 Wu et al. 2022 Wu et al. https://creativecommons.org/licenses/by/4.0/ This content is distributed under the terms of the Creative Commons Attribution 4.0 International license.

### The 230K and 326D kinase sites of MoFpk1 are essential for its biological functions.

**FIG 5:**
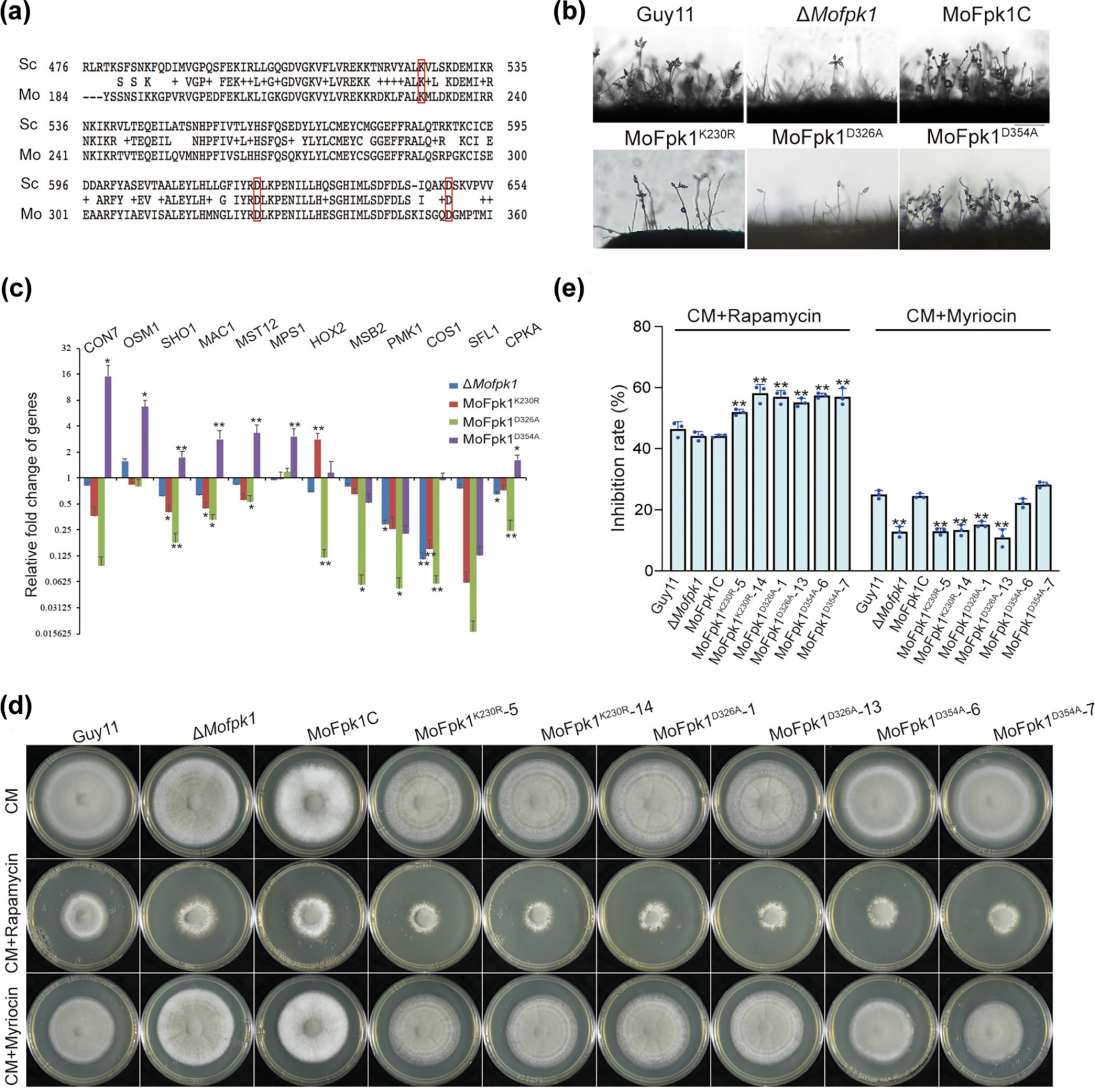
The 230K and 326D kinase sites of MoFpk1 are essential for its biological functions. (a) Alignment of conserved sequences between S. cerevisiae Fpk1 (Sc) and M. oryzae MoFpk1 (Mo). The red boxes indicate conserved amino acids. (b) Conidiophore development was observed at 24 hpi after the induction of conidia. Bar = 50 μm. (c) Expression levels of appressorium- and ALS-related genes. The expression data were normalized using the actin and 40S genes. Error bars represent the standard deviations. Data were analyzed by ANOVA using SPSS, followed by a Duncan *post hoc* LSD test for comparison (*, *P* ≤ 0.05; **, *P* ≤ 0.01). (d) Colony morphologies of the Guy11, Δ*Mofpk1*, and MoFpk1C strains grown on CM plates supplemented with rapamycin (100 ng/mL) and myriocin (1 μM) for 8 days. To ensure the accuracy of the test, two transformants were selected for each point mutant. (e) Rates of growth inhibition of each strain after 8 days [result = (diameter of the plate − diameter of the colony supplemented with rapamycin or myriocin)/diameter of the colony on the CM plate]. Data were analyzed by ANOVA using SPSS, followed by a Duncan *post hoc* LSD test for comparison (*, *P* ≤ 0.05; **, *P* ≤ 0.01). MoFpk1C, MoFpk1 complementary strain.

In the process of evolution, some kinase sites are vital for protein kinase function. After alignment with S. cerevisiae Fpk1, three amino acid sites (230K, 326D, and 354D) were screened and mutated to alanine or arginine (Fig. 5a). The MoFpk1^K230R^,MoFpk1^D326A^ strain showed growth rates, conidiation abilities, and pathogenicity similar to those of the Δ*Mofpk1* mutant. However, the MoFpk1^D354A^ strain showed similarities to Guy11 (Fig. 4a and b and Fig. 5b). The expression levels of the appressorium- and ALS-related genes showed a different trend in the MoFpk1^K230R^ and MoFpk1^D326A^ strains, similarly to the Δ*Mofpk1* strain (Fig. 5c). Also, the three site mutants showed sensitivity to rapamycin, and the MoFpk1^K230R^,MoFpk1^D326A^ strain exhibited an inhibition rate similar to that of the Δ*Mofpk1* strain when supplemented with myriocin (Fig. 5d and e). Since the pleiotropic defects arose from the substitution mutations of both 230K and 326D, we inferred that these kinase sites are essential for MoFpk1 in M. oryzae.

### MoFpk1 is involved in regulating cell wall integrity and MoMps1 phosphorylation.

**FIG 6:**
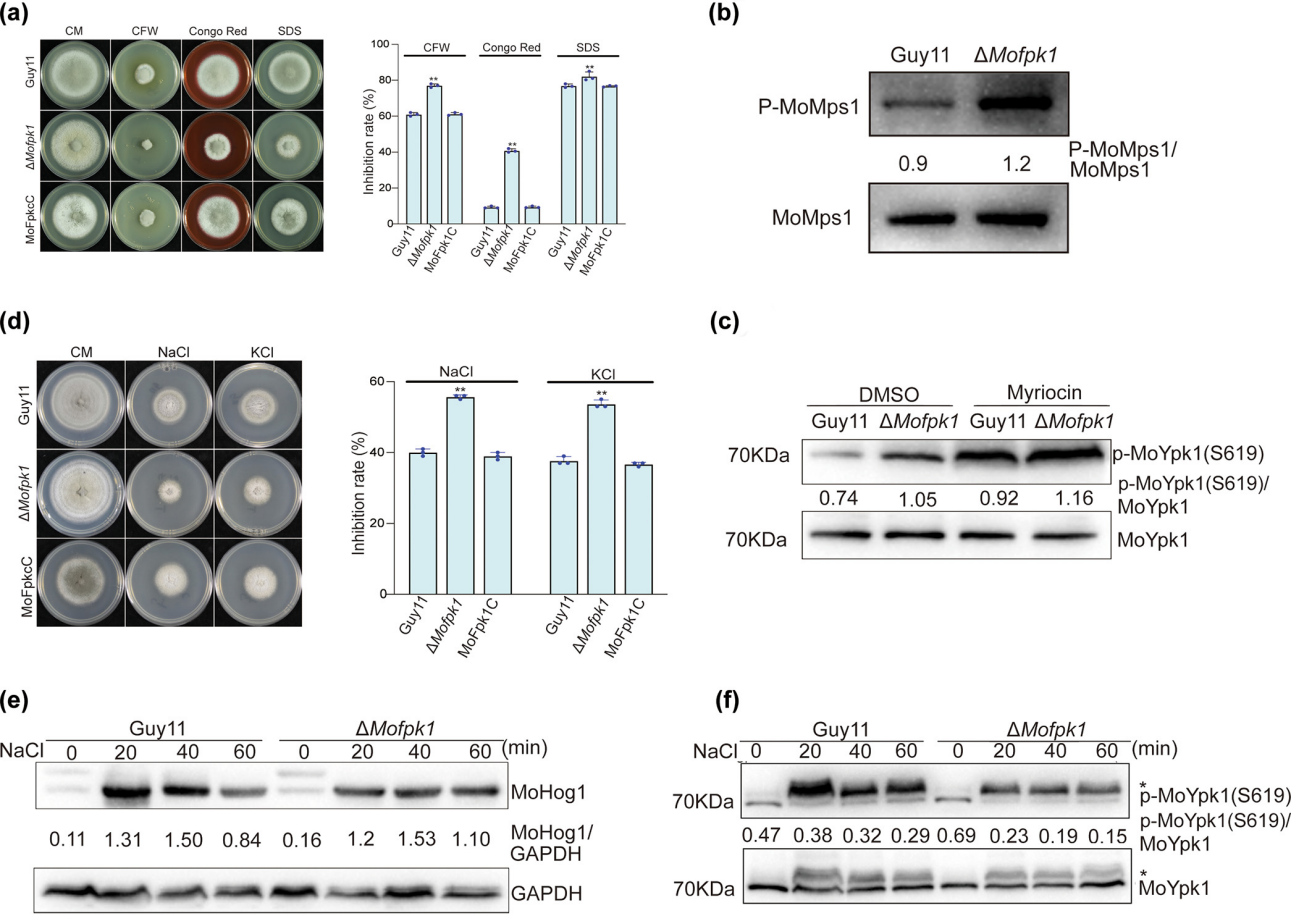
MoFpk1 is important for cell wall and plasma membrane homeostasis. (a) The Δ*Mofpk1* mutant is sensitive to cell wall stress agents. Shown are the colony morphologies of the Guy11, Δ*Mofpk1*, and MoFpk1C strains grown on medium supplemented with SDS (0.0025%), Congo red (50 μg/mL), and CFW (100 μg/mL) at 25°C for 8 days in the dark. The rates of growth inhibition of the strains on CM supplemented with different cell wall-perturbing agents were determined. Asterisks indicate a statistically significant difference. The experiment was repeated three times independently. Data were analyzed by ANOVA using SPSS, followed by a Duncan *post hoc* LSD test for comparison (*, *P* ≤ 0.05; **, *P* ≤ 0.01). (b) MoMps1 phosphorylation levels in the Δ*Mofpk1* and Guy11 strains. (c) Levels of phosphorylation of MoYpk1 in Guy11 and the Δ*Mofpk1* mutant. The strains were cultured in CM or treated with myriocin (1 μm) for 60 min before analysis. DMSO, dimethyl sulfoxide. (d) Sensitivity of the M. oryzae strains to osmotic stress. Shown are the colony morphologies of the Guy11, Δ*Mofpk1*, and MoFpk1C strains grown on medium supplemented with different osmotic stress agents, 0.5 M NaCl or 0.5 M KCl, for 8 days at 25°C. The rates of growth inhibition of the strains on medium supplemented with different osmotic stress factors were determined. The experiment was repeated three times independently. Data were analyzed as panel (a). (e) Phosphorylation of MoHog1 in Guy11 and the Δ*Mofpk1* mutant. Strains were cultured in CM for 2 days and transferred to 0.5 M NaCl for 0 min, 20 min, 40 min, and 60 min. (f) Phosphorylation of MoYpk1 when strains are treated with NaCl. Strains were culture in CM for 2 days and transferred to 0.5 M NaCl for 0 min, 20 min, 40 min, and 60 min. “*” indicates a nonspecific binding band.

To verify the role of MoFpk1 in the external stress response, the structural integrity of the cell wall was further analyzed. The Δ*Mofpk1* mutant showed obvious sensitivity to calcofluor white (CFW), Congo red, and SDS, indicating that MoFpk1 is involved in cell wall integrity (CWI) homeostasis (Fig. 6a). Numerous signaling pathways play critical roles in maintaining cell wall homeostasis. The Mps1–mitogen-activated protein kinase (MAPK) signaling pathway, homologous to the Bck1-Mkk1/Mkk2-Slt2 signaling pathway in S. cerevisiae, mainly regulates CWI in most pathogenic fungi (38). We further monitored the phosphorylation level in the Δ*Mofpk1* mutant. The deletion of *MoFPK1* increased the phosphorylation level of MoMps1 (Fig. 6b), suggesting that MoFpk1 downregulates MoMps1 phosphorylation in response to cell wall integrity.

### MoFpk1 is involved in membrane homeostasis.

Since an intermodulation mechanism between Fpk1 and Ypk1 was detected, we then monitored the phosphorylation level of MoYpk1 in the Δ*Mofpk1* mutant. The deletion of *MoFPK1* led to an increased level of phosphorylation of MoYpk1 (Fig. 6c). Next, we found that sphingolipid depletion enhanced MoYpk1 phosphorylation in both Guy11 and the Δ*Mofpk1* mutant (Fig. 6c). As for the intermodulation mechanism between MoFpk1 and MoYpk1, the enhanced phosphorylation level of MoYpk1 was more pronounced in the Δ*Mofpk1* mutant.

Two coping mechanisms (Hog1 and TORC2-Ypk1) were found to respond to hyperosmotic stress in S. cerevisiae (17, 39, 40). The Δ*Mofpk1* mutant showed greater sensitivity to the ionic imbalances caused by NaCl and KCl (Fig. 6d). Similar results were also found for the MoFpk1^K230R^,MoFpk1^D326A^ strain (Fig. S2d and e). The phosphorylation levels of MoHog1 and MoYpk1 under 0.5 M NaCl treatment were further determined. The Δ*Mofpk1* mutant and Guy11 showed a consistent upward trend in MoHog1 phosphorylation (Fig. 6e). However, the phosphorylation of MoYpk1 was decreased to a greater extent in the Δ*Mofpk1* mutant than in Guy11 (Fig. 6f). As we previously demonstrated that TORC2 activity could be determined by MoYpk1 phosphorylation (S619) (32), this indicates that hyperosmotic stress led to diminished MoTor activity, resulting in decreased MoYpk1 phosphorylation, which is independent of the MoHog1 pathway. Due to the reciprocal relationship between MoFpk1 and MoYpk1 (22), MoFpk1-dependent MoYpk1 phosphorylation showed deep attenuation in the Δ*Mofpk1* mutant, which fed back into more diminished MoTor activity in the Δ*Mofpk1* mutant.

### Effect of MoFpk1 on autophagy of M. oryzae.

**FIG 7:**
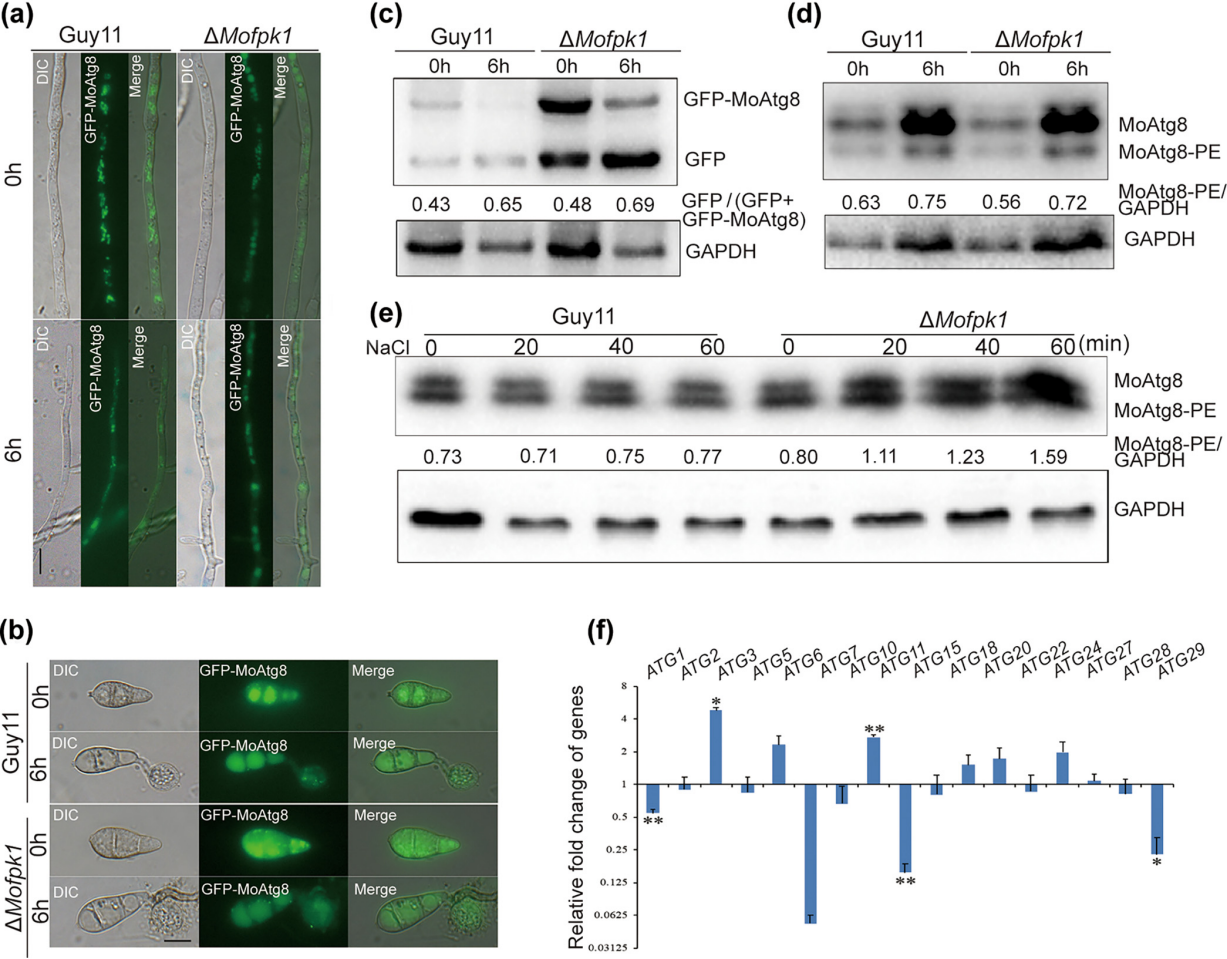
Effect of MoFpk1 on autophagy of M. oryzae. (a) Fluorescence observation of the distribution of GFP-MoAtg8 in the mycelia of Guy11 and the Δ*Mofpk1* mutant. Bar = 10 μm. DIC, differential interference contrast. (b) Fluorescence observation of the distribution of GFP-MoAtg8 in the conidia and appressoria of Guy11 and the Δ*Mofpk1* mutant. Bar = 10 μm. (c) Western blot detection of GFP-MoAtg8 degradation. The mycelium was cultured in CM and then transferred to SD−N medium for 6 h of autophagy induction. The content of GFP-MoAtg8 in the Δ*Mofpk1* mutant was significantly higher than that in the wild-type strain Guy11. (d) Detection of MoAtg8/MoAtg8-PE turnover. After being cultured in CM for 2 days, the mycelium was transferred to SD−N medium for 6 h, and the lipidation of MoAtg8 was detected by Western blotting. (e) MoAtg8 lipidation of the Δ*Mofpk1* mutant was stimulated by hyperosmotic stress. The mycelia cultured in CM for 2 days were transferred to CM containing 0.5 M NaCl for 0, 20, 40, and 60 min. (f) Relative expression levels of autophagy genes in the Δ*Mofpk1* mutant. Expression data were normalized using the actin and 40S genes. Error bars represent the standard deviations. Data were analyzed by ANOVA using SPSS, followed by a Duncan *post hoc* LSD test for comparison (*, *P* ≤ 0.05; **, *P* ≤ 0.01).

Autophagy is an evolutionarily conserved metabolic process for adaptation to various external pressure conditions. It was shown that the Δ*Mofpk1* mutant displayed green fluorescent protein (GFP)-MoAtg8 distributions (Fig. 7a and b), autophagic flux (Fig. 7c), and lipidation of autophagy (Fig. 7d) similar to those of Guy11. However, the expression levels of 17 *ATG* genes showed a different trend in the Δ*Mofpk1* mutant (Fig. 7f). Since autophagy was induced by external stress (41, 42), we then further explored the effect of external osmotic stress (Fig. 7e). The lipidation of MoAtg8 increased obviously in the Δ*Mofpk1* mutant when treated with NaCl, while there was no influence on Guy11. Hyperosmotic shock inhibited TORC2 activity by modulating PM tension in yeast (43); we inferred that MoFpk1 is important for the response to hyperosmotic stress, and the deletion of *MoFPK1* caused a decrease in MoTor activity (MoYpk1 phosphorylation), thus resulting in enhanced autophagy.

### Lipid metabolism was disturbed in the Δ*Mofpk1* mutant.

**FIG 8:**
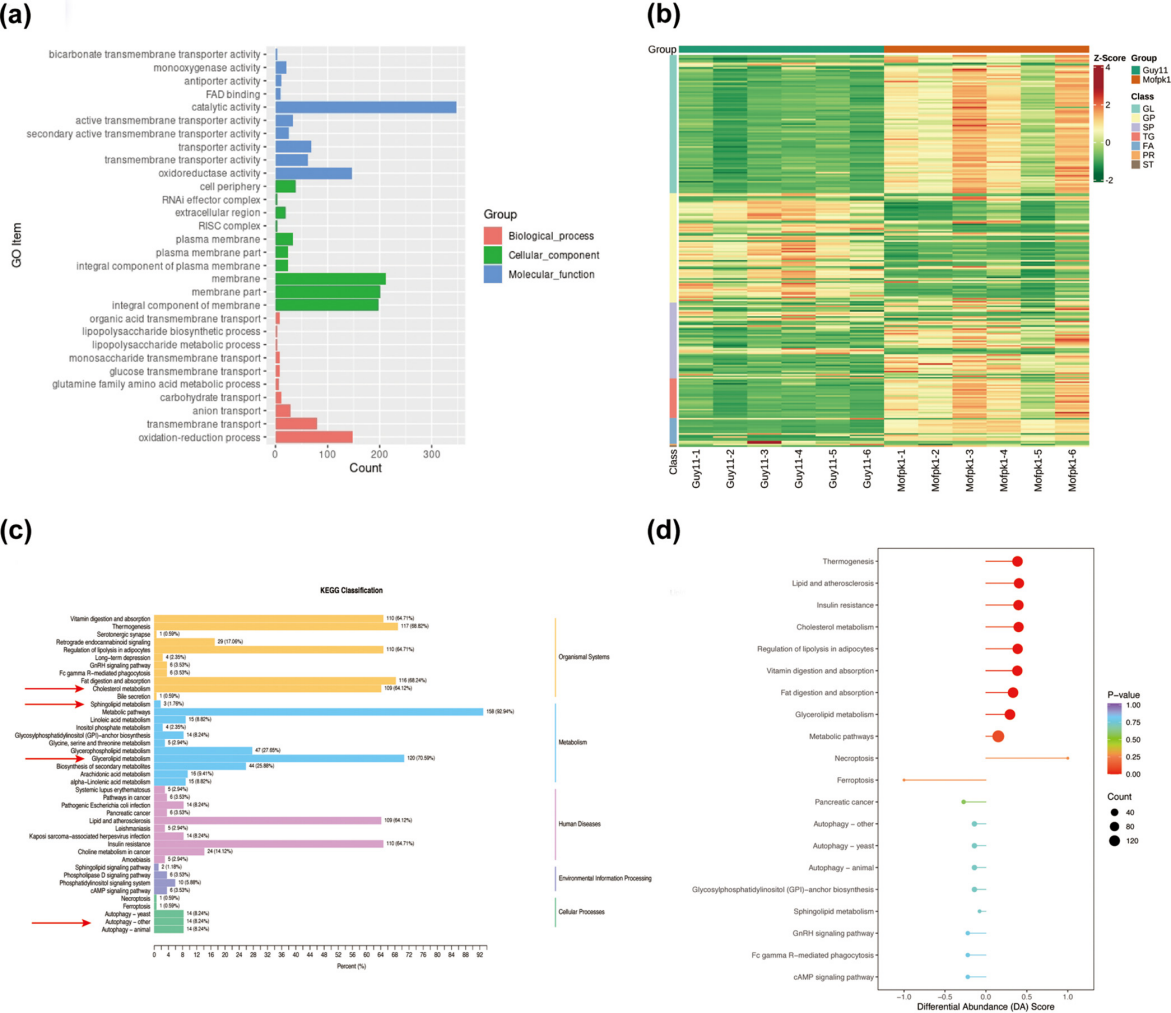
MoFpk1 is involved in lipid homeostasis. (a) Gene Ontology (GO) analysis of differentially expressed genes (DEGs) in the wild-type strain Guy11 and the Δ*Mofpk1* mutant. FAD, flavin adenine dinucleotide; RNAi, RNA interference. (b) Differential lipid clustering heat map based on the differential lipids in the Δ*Mofpk1* and Guy11 strains. The abscissa represents the sample name, the ordinate represents the different lipids, and the different colors represent the different values obtained after the normalization of different relative contents (red represents high content, and green represents low content). The different lipid classes include free fatty acids (FA), triglycerides (TG), glycerolipids (GL), sphingolipids (SP), glycerophospholipids (GP), prenol lipids (PR), and sterol lipids (ST). (c) KEGG classification of significant differences in lipid metabolism. (d) Differential abundance scores of global changes in all lipids of metabolic pathways. The length of the line segment represents the absolute value of the differential abundance score, and the size of the dots at the end of the line segment represents the number of differential lipids in the pathway. The dots are distributed on the left side of the central axis, and the longer the line segment, the more likely the overall expression of the pathway is to be downregulated, and vice versa. Line segment and dot colors reflect *P* values. GnRH, Gonadootropin-releasing hormone.

We further determined the role of MoFpk1 in lipid metabolism. The Δ*Mofpk1* mutant showed resistance to myriocin but sensitivity to amphotericin B (AmB), a polyene antifungal antibiotic that alters cell membrane permeability (Fig. 3e and f and Fig. S4a). Next, transcriptome sequencing (RNA-Seq) analysis of the Δ*Mofpk1* mutant and wild-type Guy11 was further performed (Table S2). Expression profiling identified 1,008 differentially expressed genes (DEGs), with 352 upregulated and 656 downregulated genes compared with the WT strain (*P* ≤ 0.05; log_2_ fold change [FC] of ≥1). Kyoto Encyclopedia of Genes and Genomes (KEGG) enrichment analysis and Gene Ontology (GO) enrichment analysis showed that MoFpk1 might be involved in fatty acid degradation, glycerolipid (GL) metabolism, glycosphingolipid biosynthesis, and other pathways (Fig. 8a and Fig. S5a). Next, the expression levels of 16 genes related to lipid metabolism showed a different trend in the Δ*Mofpk1* mutant compared to Guy11 (Fig. S4b). Above all, these findings indicated that MoFpk1 affected the metabolism and transport of lipids.

10.1128/mbio.02279-22.4 FIG S4 MoFpk1 is involved in lipid metabolism. (a) Rate of growth inhibition by amphotericin B. Data were analyzed by ANOVA using SPSS, followed by a Duncan *post hoc* LSD test for comparison, and the values are statistically significant when the *P* values were ≤0.05 (*) and ≤0.01 (**). (b) The expression levels of lipid metabolism-related genes were confirmed by qRT-PCR. Actin and 40S genes were used for calibration against the profiles of the Guy11 and Δ*Mofpk1* strains. Error bars represent the standard deviations. Data were analyzed by ANOVA using SPSS, followed by a Duncan *post hoc* LSD test for comparison (*, *P* ≤ 0.05; **, *P* ≤ 0.01). (c) Orthogonal projections to latent structures discriminant analysis (OPLS-DA) results showed a lipidome separation trend between the Guy11 and Δ*Mofpk1* strains by performing principal-component analysis on the samples. (d) Effects of rapamycin and myriocin on the lipid metabolism and transport of the Guy11 and Δ*Mofpk1* strains (Bar = 10 μm). Download FIG S4, JPG file, 1.2 MB. Copyright © 2022 Wu et al. 2022 Wu et al. https://creativecommons.org/licenses/by/4.0/ This content is distributed under the terms of the Creative Commons Attribution 4.0 International license.

10.1128/mbio.02279-22.5 FIG S5 MoFpk1 may be involved in autophagy by regulating lipid metabolism. (a) KEGG terms of the Δ*Mofpk1* mutant. KEGG analysis shows significantly different processes. (b) Differential lipid clustering heat map based on the differential lipids in Guy11 and Guy11 treated with 50 ng/mL rapamycin. The abscissa represents the sample name, the ordinate represents the different lipids, and the different colors represent the different values obtained after the normalization of the different relative contents (red represents high content, and green represents low content). Free fatty acids (FA), triglycerides (TG), glycerolipids (GL), sphingolipids (SP), glycerophospholipids (GP), prenol lipids (PR), and sterol lipids (ST) are shown. (c) Differential lipid clustering heat map based on the differential lipids in the Δ*Mofpk1* mutant and the Δ*Mofpk1* mutant treated with 50 ng/mL rapamycin. (d) Differential lipid clustering heat map based on the differential lipids in Guy11 treated with 50 ng/mL rapamycin and the Δ*Mofpk1* mutant treated with 50 ng/mL rapamycin. Download FIG S5, TIF file, 2.1 MB. Copyright © 2022 Wu et al. 2022 Wu et al. https://creativecommons.org/licenses/by/4.0/ This content is distributed under the terms of the Creative Commons Attribution 4.0 International license.

10.1128/mbio.02279-22.7 TABLE S2 DEGs in wild-type Guy11 and the Δ*Mofpk1* mutant. Download Table S2, XLSX file, 0.1 MB. Copyright © 2022 Wu et al. 2022 Wu et al. https://creativecommons.org/licenses/by/4.0/ This content is distributed under the terms of the Creative Commons Attribution 4.0 International license.

To further determine the role of MoFpk1 in lipid metabolism, widely targeted metabolome analysis between the Δ*Mofpk1* mutant and Guy11 was performed using a liquid chromatography-electrospray ionization-tandem mass spectrometry (LC-ESI-MS/MS) system. Orthogonal projections to latent structures discriminant analysis (OPLS-DA) showed a trend of lipidome separation between Guy11 and the Δ*Mofpk1* mutant (Fig. S4c), in which 258 differences in lipids between Guy11 and the Δ*Mofpk1* mutant were preliminarily screened (variable importance in projection [VIP] value of ≥1; fold change of ≥2 and ≤0.5). (Table S3). Next, a differential lipid clustering heat map was drawn. Compared to Guy11, free fatty acids (FAs), triglycerides (TGs), glycerolipids (GLs), and sphingolipids (SPs) were significantly upregulated, while glycerophospholipids (GPs) were downregulated markedly (Fig. 8b). Consistent with previous observations of decreased lipid droplet metabolism in the Δ*Mofpk1* mutant (Fig. S3a and b), the deletion of *MoFPK1* showed severely disrupted lipid homeostasis. Next, we mapped the landscape of metabolic-transcriptional alterations in the context of MoFpk1. As shown in Fig. 8c and d, autophagy, glycerol lipid metabolism, and sphingolipid synthesis were significantly modulated by MoFpk1. The deletion of MoFpk1 resulted in the inability of the flippase complex to maintain the normal turnover of PE and PS, which are members of the GPs (Table S3), thus resulting in membrane asymmetry defects and triggering a series of lipid metabolism disorders.

10.1128/mbio.02279-22.8 TABLE S3 Differential lipids in different strains and different treatments. (1) Differences in lipid metabolites between wild-type Guy11 and the Δ*Mofpk1* mutant. (2) Differences in lipid metabolites between wild-type Guy11 and wild-type Guy11 treated with rapamycin. (3) Differences in lipid metabolites between the Δ*Mofpk1* mutant and the Δ*Mofpk1* mutant treated with rapamycin. (4) Differences in lipid metabolites between wild-type Guy11 treated with rapamycin and the Δ*Mofpk1* mutant treated with rapamycin. Download Table S3, XLSX file, 0.2 MB. Copyright © 2022 Wu et al. 2022 Wu et al. https://creativecommons.org/licenses/by/4.0/ This content is distributed under the terms of the Creative Commons Attribution 4.0 International license.

To further explore the relationship between autophagy and lipid metabolism, the Δ*Mofpk1* mutant and Guy11 were inoculated into complete medium (CM) supplemented with rapamycin or myriocin. Compared with Guy11, lipid droplets exhibited large and numerous fusions in the Δ*Mofpk1* mutant when treated with rapamycin. However, when treated with myriocin, the number of small lipid droplets in the Δ*Mofpk1* mutant increased significantly (Fig. S4d). We speculate that there may be an unknown pattern in M. oryzae, that is, that MoFpk1 participated in the homeostasis of autophagy and lipid metabolism.

To further explore the interplay between lipid metabolism and autophagy, widely targeted metabolome analyses between the Δ*Mofpk1* mutant treated with rapamycin and the wild-type Guy11 strain treated with rapamycin were performed. When autophagy is induced, GPs, SPs, GLs, FAs, TGs, and PRs (prenol lipids) are significantly downregulated in both wild-type Guy11 and the Δ*Mofpk1* mutant compared to untreated conditions (Fig. S5b and c and Table S3). We deduced that autophagy led to the downregulation of lipid metabolism. However, in addition to an interesting lipid, GP, which was downregulated regardless of rapamycin treatment or the deletion of *MoFPK1*, most lipids were upregulated in the Δ*Mofpk1* mutant both under normal conditions and with autophagy induction (Fig. S5d and Table S3). Overall, autophagy induction was accompanied by the severe downregulation of lipid metabolism, and GP appeared to play a particularly pronounced role in the link between autophagy and lipid metabolism.

## DISCUSSION

**FIG 9:**
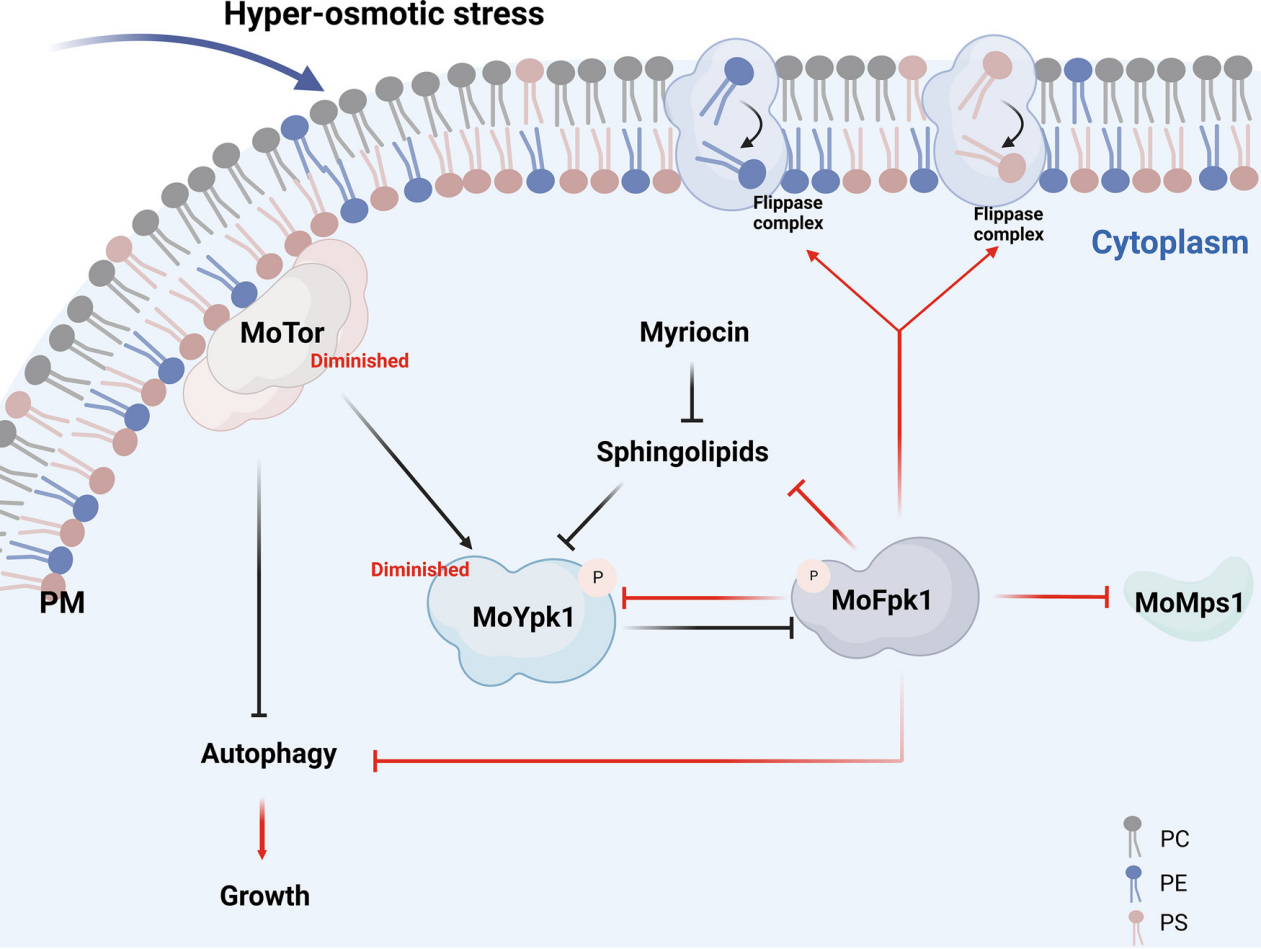
Model of the involvement of MoFpk1 in lipid metabolism, autophagy, and hyperosmotic stress. MoFpk1 is a protein that regulates lipid asymmetry and maintains cell wall and plasma membrane homeostasis. During cell wall stress, MoFpk1 participates in cell wall integrity by regulating MoMps1; MoFpk1 inhibits MoYpk1 phosphorylation and sphingolipid synthesis. Moreover, the synthesis defect of sphingolipids leads to an enhanced level of MoYpk1 phosphorylation. When treated with NaCl, hyperosmotic stress causes diminished MoYpk1 phosphorylation and MoTor activity and thus results in enhanced autophagy in the Δ*Mofpk1* mutant. The deletion of MoFpk1 results in PE and PS turnover defects and a series of lipid metabolism disorders. Under hyperosmotic stress, the membrane lipid asymmetry defect leads to altered membrane tension and MoYpk1 phosphorylation and downregulated MoTor activity, thus leading to enhanced autophagy. The red lines represent the results of this study, and the black lines represent the results of previous studies. PC, Phosphatidyl cholines.

During eukaryotic evolution, the TOR-AGC kinase signaling module is involved in the coordinated regulation of cell growth and survival. In this study, we systematically identified 20 members of the AGC family and characterized their pleiotropic roles in the genetics and biological functions of M. oryzae. We integrated previous findings and our key findings and found that AGC kinases were involved in growth (8 genes), conidiation (13 genes), conidial germination (9 genes), appressorium formation (9 genes), and pathogenicity (5 genes). Interestingly, after genome-wide and biological analyses, we also identified an AGC family member, MoFpk1, that plays an indispensable role in the development, hyperosmotic stress, autophagy, and lipid metabolism of M. oryzae (Fig. 9).

Throughout evolution, organisms have developed many mechanisms to adapt to novel environments, and the evolutionary adaptation of proteins is integral to these mechanisms. As a result, kinases have undergone many lineage-specific expansions and reductions. The TOR-AGC kinase signaling module is conserved during eukaryotic evolution (44). In the evolutionary core of TOR and the TOR pathway, the duplications of ancestral AGC kinases have promoted the increasing complexity of TOR and, among these duplication events, gave rise to the S6K, RSK, and PKB subfamilies (30), in which many AGC subfamilies are functionally and genetically linked to TOR and participate in many processes such as sphingolipid biosynthesis and membrane lipid distribution (22, 45–47). The duplications and subfunctionalizations of some AGC kinases shaped the complexity of the TOR pathway (30), and the distinct TOR complexes account for the diverse, specific, and selective rapamycin inhibition of TOR signaling (48), which explains the phenotypic diversity of AGC kinase mutants, suggesting that the AGC kinases are both highly conserved and flexible among M. oryzae strains (Fig. 1 to 3).

MoFpk1 is a member of the RSK subfamily of the AGC kinase family (Fig. 1). Deficiencies in flippase activity cause many abnormalities in normal metabolic activity, such as endocytosis defects (49) and Golgi complex and endosomal vacuolar system trafficking defects (50), etc. TORC2 regulates cell membrane tension (for instance, sphingolipid depletion and hyperosmotic stress), involving the level of phosphorylation of the downstream substrate protein Ypk1 (51). Ypk1 regulates the biosynthesis of sphingolipid by the phosphorylated palmitoyl-CoA transferase inhibitors Orm1 and Orm2 and is involved in plasma membrane lipid homeostasis by phosphorylated Fpk1 (16). In addition, hyperosmotic stress leads to a rapid and remarkable decrease in Ypk1 phosphorylation at its TORC2 site (16), which may result from phosphatase activation, TORC2 catalytic activity inhibition, or both (17). However, the underlying mechanisms remain largely vague between Ypk1, Fpk1, as well as membrane stresses (22, 52). Our results suggested that the deletion of MoFpk1 led to developmental defects in M. oryzae (Fig. 4). MoFpk1 phosphorylation inhibited MoYpk1, and sphingolipid depletion led to the activation of MoTor activity (MoYpk1 phosphorylation) (Fig. 6c). We also provide a novel mechanism for sensing and responding to certain membrane stresses; that is, MoFpk1 participates in autophagy by maintaining MoYpk1 phosphorylation and MoTor activity under hyperosmotic stress.

The PM forms selective osmotic barriers and dynamic interfaces, which correlate with the content and distribution of lipids in the cell. Maintaining membrane homeostasis is important for cellular stress responses such as external osmotic stress or internal metabolic processes (e.g., autophagy) (32). Recently, new roles of various membrane lipids in autophagosome formation are emerging. Some Atg proteins transfer lipids between membranes and are thus involved in the regulation of lipid metabolism (53). For instance, Atg2 can bind tens of GPs simultaneously and transfer lipids robustly *in vitro* (54). Interestingly, some membrane lipids (e.g., sterols and sphingolipids) are involved in PM tension regulation by controlling nitrogen signaling, thus leading to TOR activation (55). We previously demonstrated that a sterol-trafficking-related protein, MoVast1, actively regulates MoTor activity and participates in autophagy by sterol maintenance for PM tension adjustment (32). These results suggested that PM lipid homeostasis is also involved in TOR signaling. As TOR activity has been reported to be a switch involved in the initiation and termination of autophagy (32, 56), PM lipid homeostasis and autophagy are coordinated to maintain normal cellular metabolism. In this study, we found that autophagy induction led to the downregulation of many lipids (see Fig. S5b to d in the supplemental material), suggesting that the lipid content appears to be a marker of autophagy, and we identified a novel flippase-activating kinase, MoFpk1, in M. oryzae and revealed novel aspects of MoFpk1 in lipid homeostasis regulation in the PM. The deletion of MoFpk1 leads to PE and PS turnover defects (Table S3) and a series of lipid disorders (Fig. 3e and f, Fig. 8b, and Fig. S4a). We inferred that MoFpk1 participated in autophagy by membrane asymmetry and MoTor activity maintenance under hyperosmotic stress.

In conclusion, this study has revealed the important role of AGC kinases in M. oryzae. We also found an interesting AGC kinase, MoFpk1, that plays an indispensable role in osmotic regulation, lipid homeostasis, and autophagy. This study provides a new mechanism for the internal linkage between lipid metabolism and autophagy, which may help in the development of new fungicide targets for controlling this devastating disease.

## MATERIALS AND METHODS

### Fungal strains and culture conditions.

The Guy11 strain (a gift from Talbot Laboratory, UK) and the mutants of M. oryzae used in this experiment were cultured on a complete medium (CM) plate at 25°C with a photoperiod of 16 h of light/8 h of darkness for 8 days, as previously reported (32, 57–59).

### Gene knockout and complementation assays.

The high-throughput gene knockout system was generated as previously reported (32, 57–59), with slight modifications. The 1.5-kb upstream fragment (UF) or downstream fragment (DF) of the AGC kinases was amplified by PCR using Phanta Max superfidelity DNA polymerase (Vazyme Biotech Co., Ltd., Nanjing, China) (primers are listed in Table S1 in the supplemental material). The pKO1B or pKO3A vector was digested with *Xba*I and *Hin*dIII. The hygromycin B phosphotransferase (HPH) gene was amplified from the pCB1003 vector and fused with the linearized vector, the UF, and the DF to generate a gene deletion cassette, which was further verified using Green *Taq* mix (Vazyme Biotech Co., Ltd., Nanjing, China). Next, the gene deletion cassette was transferred to Guy11 using the Agrobacterium tumefaciens-mediated transformation (ATMT) method. Transformants were selected on CM with 200 μg/mL HPH. For the complementary assay, pKD5-GFP was digested with *Bam*HI, fused with the AGC kinase fragments without the stop codon (TAA), and then transformed into mutants using the ATMT method. Transformants were selected on CM with 200 μg/mL sulfonylurea (SUR). The complementary strains were also used for subcellular localization analysis.

10.1128/mbio.02279-22.6 TABLE S1 Primers used in this study. Download Table S1, DOCX file, 0.02 MB. Copyright © 2022 Wu et al. 2022 Wu et al. https://creativecommons.org/licenses/by/4.0/ This content is distributed under the terms of the Creative Commons Attribution 4.0 International license.

### Phenotypic analysis of M. oryzae mutants.

To determine the relative growth rate of M. oryzae, 5-mm agar blocks of strains were cut and inoculated onto CM plates. The colony diameters were measured and photographed at 8 days postinoculation (dpi). Three replicates were performed for each experiment. For the conidiation analysis, conidia were harvested with sterile water and further counted with a hemocytometer under a microscope (32, 57–59). For the observation of conidiophores, the mycelium of strains was scraped and placed in a ventilated place to dry for about 1 h. The edge was then cut off, incubated in the dark, and then observed and photographed under a microscope after 24 h (32, 57–59). For the pathogenicity analysis, the plants used were rice CO-39 and barley ZJ-8. M. oryzae strains were placed onto 2-week-old detached barley or rice leaves and observed and photographed at 72 hpi. For the spray assay, conidia were collected and diluted to 5 × 10^4^ to 1 × 10^5^ conidia/mL with a 0.2% gelatin solution, sprayed onto the rice seedlings using an artist’s airbrush, placed in the dark for 48 h, and then transferred to 16 h of light/8 h of darkness at 25°C for 8 days. For the infection assay, 5-mm mycelial blocks were placed onto detached barley leaves. At 24 hpi, 48 hpi, 72 hpi, and 96 hpi, the leaves were decolorized and observed under a microscope (32, 57–59). For the induction of appressorium formation, the conidia on the CM plates were washed and diluted to 5 × 10^4^ conidia/mL. A 20-μL conidial suspension was dropped onto a plastic film, placed in a humid box, cultured in the dark, and observed at 4 h, 8 h, 16 h, 24 h, and 48 h. For the induction of ALS formation, the strains cultured in liquid CM were dropped onto a hydrophobic membrane or rice leaves for 48 h. For glycogen and lipid droplet staining, the conidia and appressorium were stained with KI (potassium iodide)/I_2_(iodide) or Bodipy (1 μg/mL) (catalog number D-3922; Invitrogen, Carlsbad, CA, USA) (32, 57–59).

### Growth inhibition assay.

For stress assays, 100 ng/mL rapamycin (catalogue number 53123-88-9; Sigma-Aldrich, Germany), 1 μM myriocin (catalogue number HY-N6798; MCE, NJ, USA), 100 μg/mL CFW, 50 μg/mL Congo red, 0.0025% SDS, 0.5 M NaCl, 0.5 M KCl, and 1 M sorbitol were added to CM. For carbon source utilization, minimal medium (MM) was supplemented with 1% glucose, 1% sucrose, 1% xylose, 1% maltose, 1% olive, 1% Tween 80, and 50 mM sodium acetate (NaAc) as different carbon sources. At 8 dpi, the colony diameters of the strains were measured and photographed.

### Phosphorylation level assay.

Anti-phospho-p44/42 MAPK antibody (catalogue number 9101S; Cell Signaling Technology, Boston, MA, USA) was used to determine the MoMps1 phosphorylation level. The anti-phospho-MoYpk1 (S619) antibody and the anti-MoYpk1 antibody (prepared by ABclonal Biotechnology Co., Ltd., Wuhan, China) were used to detect MoYpk1 phosphorylation (32). The anti-MAPK14 (T180/Y182) antibody (catalogue number 9211S; Cell Signaling Technology, Boston, MA, USA) was used for MoHog1 phosphorylation detection. Anti-glyceraldehyde-3-phosphate dehydrogenase (GAPDH) was used as a control. ImageJ software was used to analyze the level of phosphorylation.

### Autophagy assay.

After culture in liquid CM, the strains were transferred to synthetic defined medium without amino acids and ammonium sulfate (SD−N) for 3 or 6 h. GFP-MoAtg8 was detected using a GFP probe (catalogue number ab32146; Abcam, Shanghai, China). For MoAtg8 lipidation assays, after being transferred to SD−N medium or CM containing 0.5 M NaCl, the immunoblots (13.5% SDS-PAGE gel in the presence of 6 M urea) of MoAtg8-PE were probed with anti-Atg8 (catalogue number PM090; BML Beijing Biotech, Beijing, China) (32). The hyphae were photographed with a microscope (Eclipse 80i 100× oil; Nikon, Japan) after 0 and 6 h of induction.

### Transcription and qRT-PCR analyses.

The RNA was extracted by the TRIzol method using the RNA 6000 Nano kit (catalogue number 5067-1511; Agilent, Shanghai, China) and analyzed on the Illumina (San Diego, CA, USA) HiSeq 2500 platform. Differentially expressed gene analysis was performed using the DESeq R package (https://www.rdocumentation.org/packages/DESeq2/versions/1.12.3), and the differentially expressed genes were further validated by qRT-PCR (32). The PrimeScript RT reagent kit with gDNA Eraser (catalogue number RR047B; TaKaRa, Japan) or the RevertAid first-strand cDNA synthesis kit (catalogue number K1622; Thermo Scientific, MA, USA) was used for the reverse transcription of RNA according to the operating instructions. Three biological replicates of each experiment were conducted.

### Bioinformatics analysis method.

The AGC kinases of M. oryzae were identified using Kionmer v1.0 (http://www.compbio.dundee.ac.uk/kinomer/index.html) (25). The kinase sequences of M. oryzae and S. cerevisiae AGC kinases obtained from the NCBI (https://ncbi.nlm.nih.gov) were used to construct the phylogenetic tree using MEGA11 (maximum likelihood method with 1,000 bootstraps, the Poisson correction model, and partial deletion) (60). The Ensembl project (https://www.ensembl.org) (31) was used to draw AGC kinase physical locations; Map Gene 2 Chromosome v2 (MG2C) online software (http://mg2c.iask.in/mg2c_v2.0/) was used to map the chromosomes. Exon/intron structures were determined by TBtools (29). MEME (Multiple Em for Motif Induction) (61) was used to predict putative motifs (http://meme-suite.org/). TBtools (29) was used for the combination.

### Widely targeted metabolomic profiling.

After the strains were grown in liquid CM for 2 days, 100 ng/mL rapamycin was added to induce autophagy for 6 h at 25°C at 200 rpm. The extensively targeted metabolomic analyses of the Δ*Mofpk1* mutant and wild-type strain Guy11 were performed as previously reported (32, 62). Sample extracts were analyzed using an LC-ESI-MS/MS system (ultraperformance liquid chromatography [UPLC]) (Shim-pack UPLC CBM20A system; Shimadzu, Japan).
