# Supplementary figures and images for: Genome-Wide Analysis of AGC Kinases Reveals that MoFpk1 Is Required for Development, Lipid Metabolism, and Autophagy in Hyperosmotic Stress of the Rice Blast Fungus Magnaporthe oryzae
Source: mBio. 2022 Oct 19;13(6):e02279-22. doi: 10.1128/mbio.02279-22 (PMC9765699; doi:10.1128/mbio.02279-22)

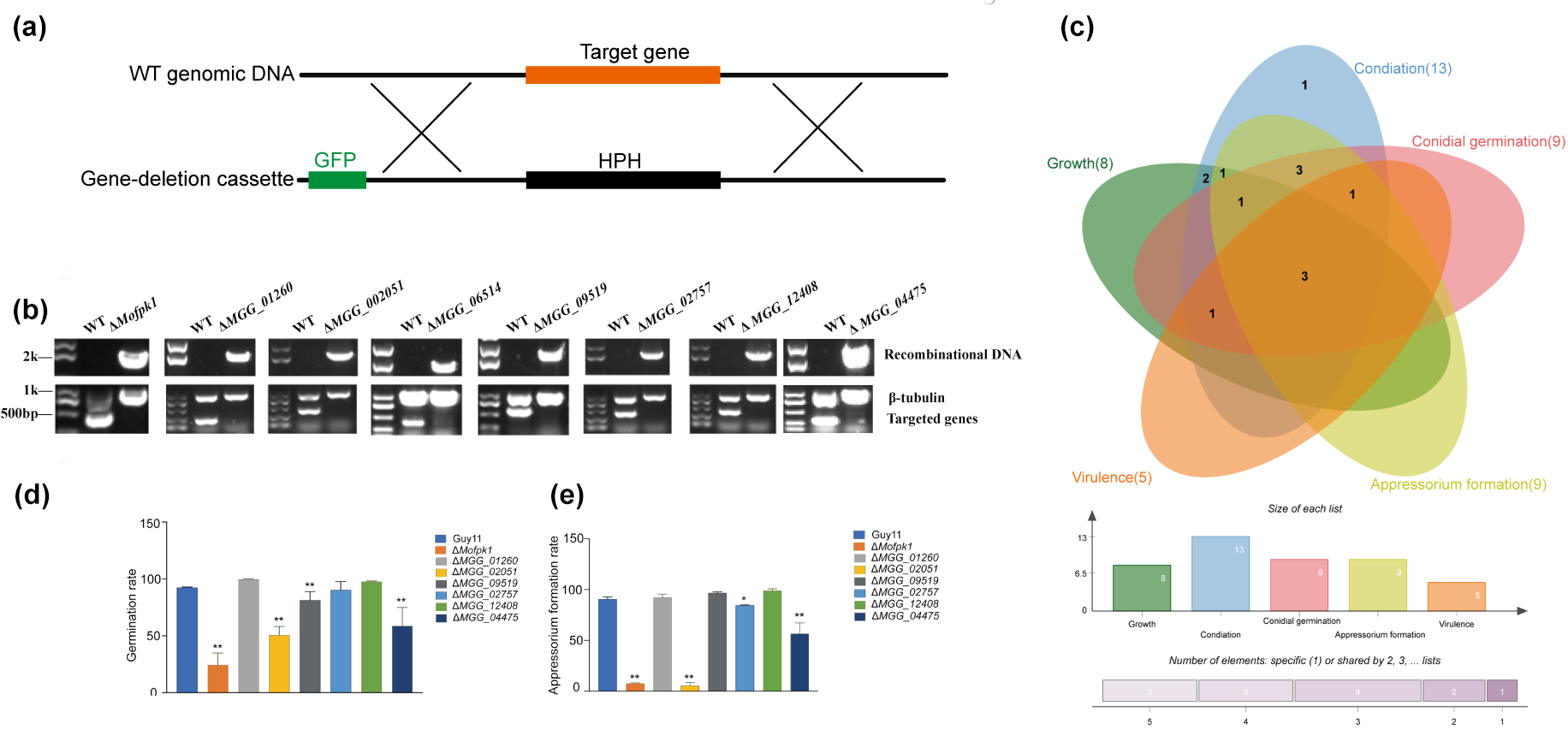

Supplement: FIG S1 [file mbio.02279-22-s0001.tif]

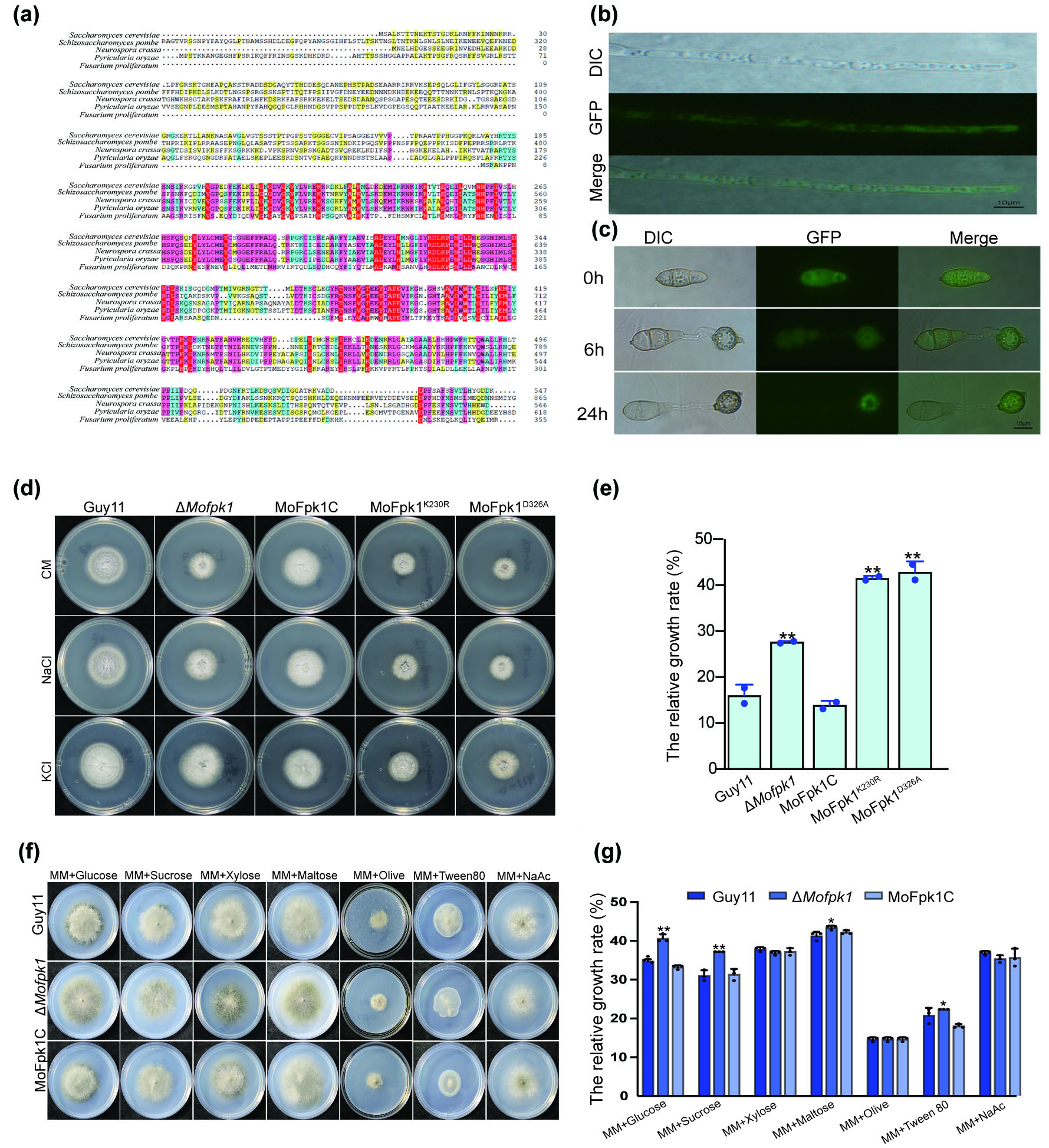

Supplement: FIG S2 [file mbio.02279-22-s0002.jpg]

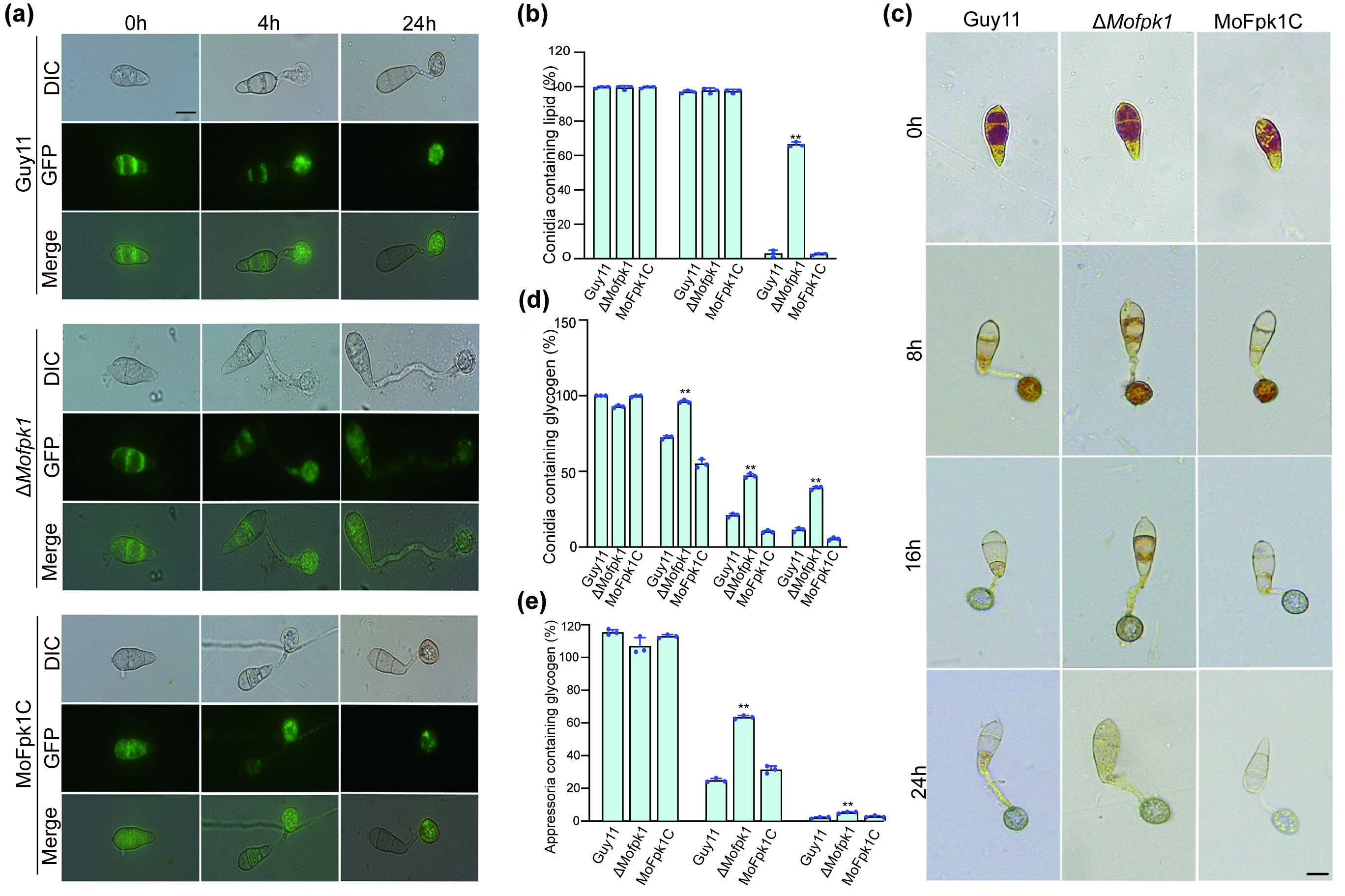

Supplement: FIG S3 [file mbio.02279-22-s0003.jpg]

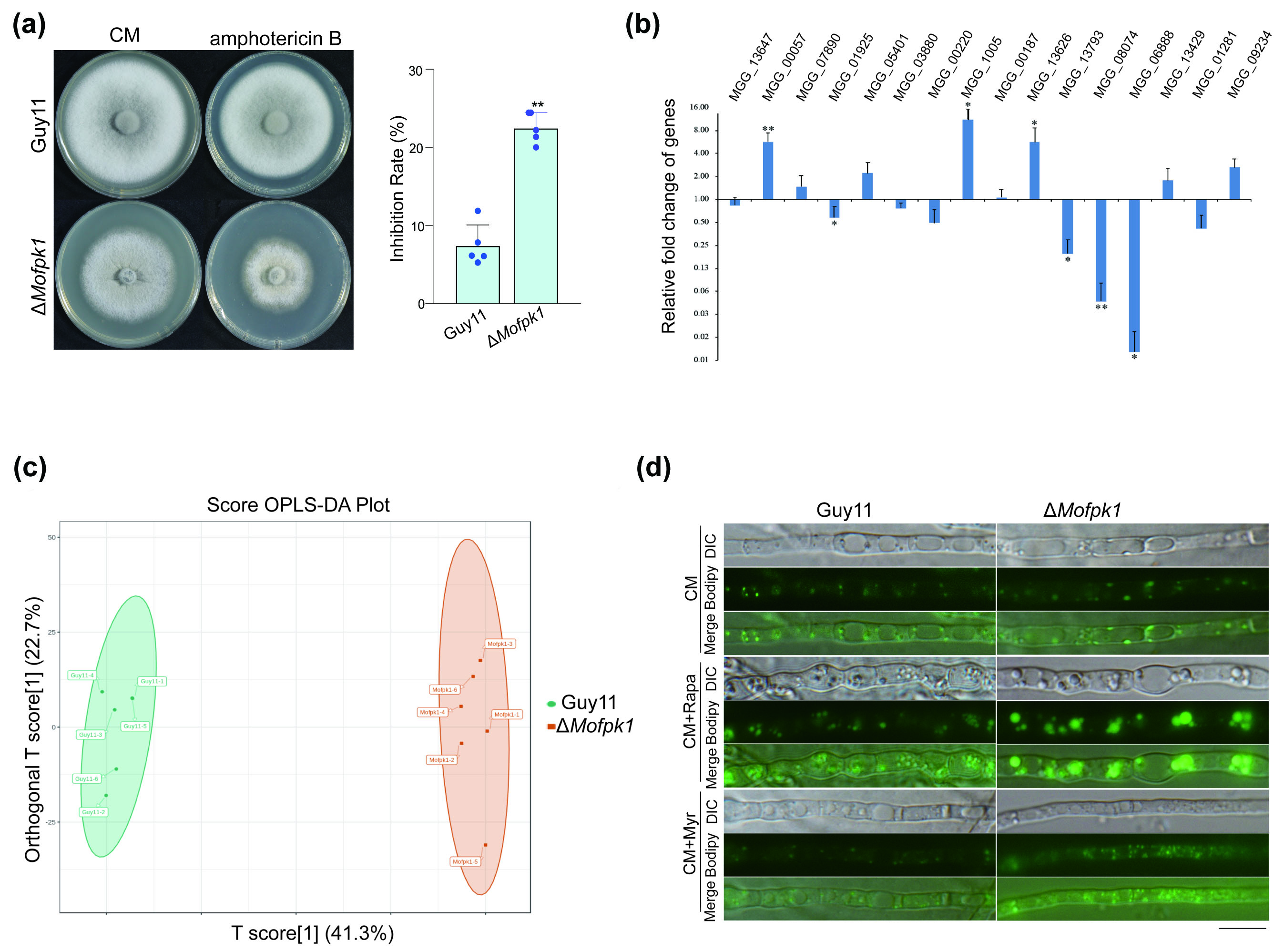

Supplement: FIG S4 [file mbio.02279-22-s0004.jpg]

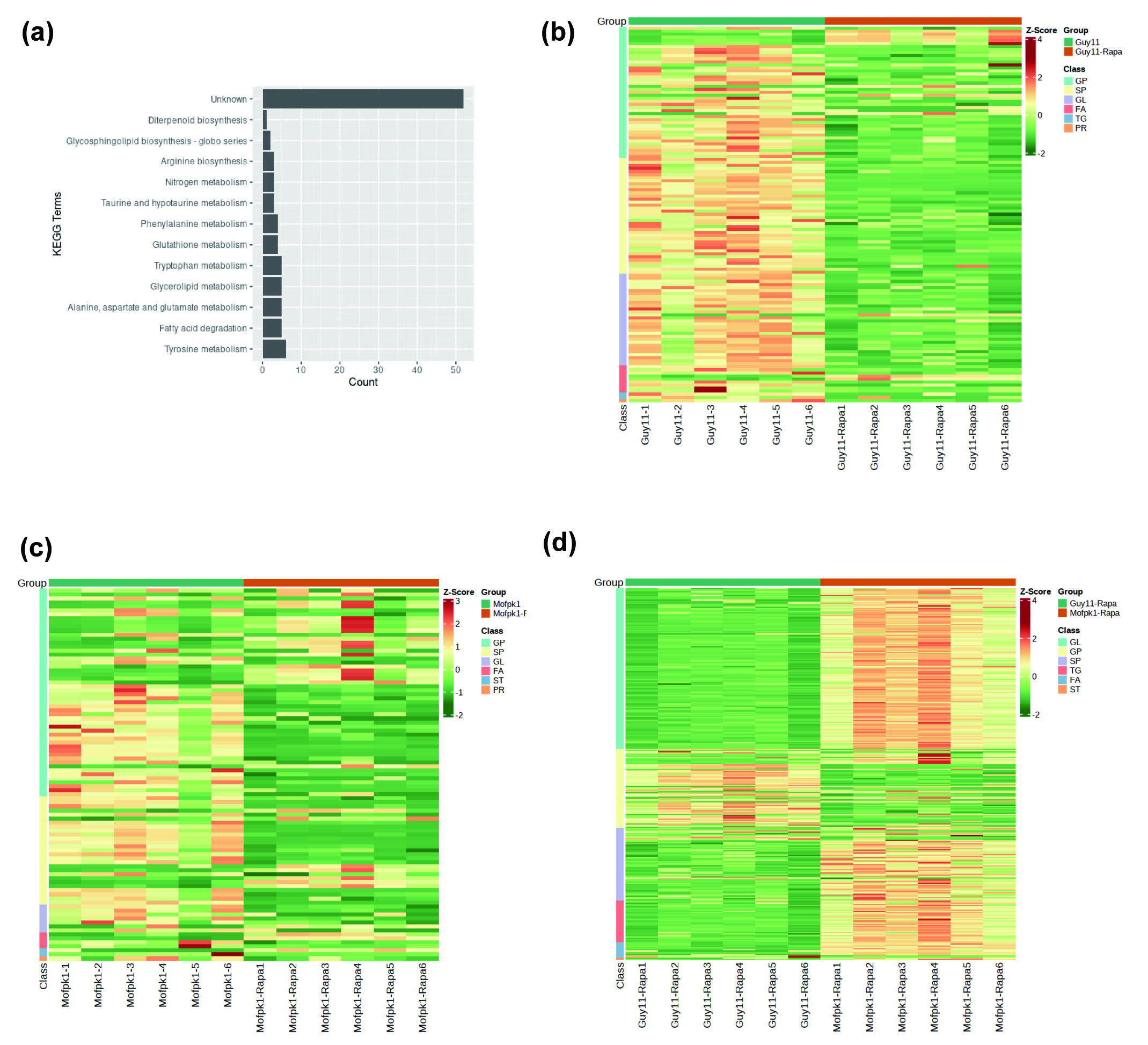

Supplement: FIG S5 [file mbio.02279-22-s0005.tif]
