# Supplementary material for: Genome-Wide Analysis of AGC Kinases Reveals that MoFpk1 Is Required for Development, Lipid Metabolism, and Autophagy in Hyperosmotic Stress of the Rice Blast Fungus Magnaporthe oryzae
Source: mBio. 2022 Oct 19;13(6):e02279-22. doi: 10.1128/mbio.02279-22 (PMC9765699; doi:10.1128/mbio.02279-22)
Supplement: TABLE S1 [file mbio.02279-22-s0008.docx]

**S1 Table.** Primers used in this study.

| Primer name | Primer |
| --- | --- |
| MGG_07012 Up F | GGTACCCGGGGATCCTCTAGACCGTAAGTTCAGGGTCATCATC |
| MGG_07012 Up R | CTCCTTCAATATCATCTTCTAGATGCGGCGATGTCTCC |
| MGG_07012 Down F | TGCCCGTCACCGAGATTTAGTCGTCTGCGGAGTCTGTC |
| MGG_07012 Down R | ACGACGGCCAGTGCCAAGCTTATGAAGAGTTGGTGGGATGAC |
| MGG_07012 Inner F | CACACTGCTAAATGCCGAGA |
| MGG_07012 Inner R | GCACAGTGTTGCTGTGGACT |
| MGG_07012 Long F | GAACAAGTGGCGTACAGACG |
| MGG_12408 Up F | GTACCCGGGGATCCTCTAGACGGAGCGTTTGAAATGTG |
| MGG_12408 Up R | CATTCATTGTTGACCTCCACTATTTGTGACGGTTGTGAGC |
| MGG_12408 Down F | GGGCAAAGGAATAGAGTAGATGGGACGCTGGACTATCTGC |
| MGG_12408 Down R | CGACGGCCAGTGCCAAGCTTTATGGCGGTGTTATCTGG |
| MGG_12408 Inner F | CGTGCAGGAACTCCCGAAT |
| MGG_12408 Inner R | GGCGATGGCAACGATGAT |
| MGG_12408 Long F | CGGAGCGTTTGAAATGTG |
| MGG_02757 Up F | GGTACCCGGGGATCCTCTAGAGAGGTCGGAGGAGGTCAAG |
| MGG_02757 Up R | CTCCTTCAATATCATCTTCTTAGTATATGCTGTCGGTTGGTG |
| MGG_02757 Down F | TGCCCGTCACCGAGATTTAGCACTCTCAGCCACCACATTAC |
| MGG_02757 Down R | ACGACGGCCAGTGCCAAGCTTCGTTCAGCAGGTTCATTCATTC |
| MGG_02757 Inner F | CTCAGGGATAGACCGACGAC |
| MGG_02757 Inner R | TTGCTCATGACCTTGAGTGC |
| MGG_02757 Long F | GCTCCACTCCTTCCATCCAG |
| MGG_09519 Up F | GTACCCGGGGATCCTCTAGATTGTTTCTTACCGAGGAC |
| MGG_09519 Up R | CATTCATTGTTGACCTCCACTAATGGATGAACCGATGTGA |
| MGG_09519 Down F | GGGCAAAGGAATAGAGTAGATG ATTTATAGACTTAGGTGCTG |
| MGG_09519 Down R | CGACGGCCAGTGCCAAGCTT CGCTGTGAAGCCGAGGAA |
| MGG_09519 Inner F | CATCAAGCCAGACAACATAC |
| MGG_09519 Inner R | CAGGCTATCAGAGGGAAGT |
| MGG_09519 Long F | CGGTCTACGCATTGCCTT |
| MGG_01260 Up F | GGTACCCGGGGATCCTCTAGACATTGGCGGTGATACATT |
| MGG_01260 Up R | ATTCATTGTTGACCTCCACTATGATACCCTGTCACTTTCG |
| MGG_01260 Down F | GGCAAAGGAATAGAGTAGATGAAGCGTGGGACGGATAAT |
| MGG_01260 Down R | ACGACGGCCAGTGCCAAGCTTAAAGGCTGCGTTTGTTGT |
| MGG_01260 Inner F | AACCTCGCCGTTTCCTTT |
| MGG_01260 Inner R | CAGAATAACCGTGCCGTAG |
| MGG_01260 Long F | AGACGAATGGGAGGAAAT |
| MoFpk1C F | AGGAATAGAGTAGATGGAATTCGTCACGAACGAGGCAAAG |
| MoFpk1C R | TAGACTTCCCGGGGATGGATCCTCTCCACGATGTCCAACT |
| MoFpk1(site mutate) UP F | CCCGGGGATCCTCTAGAACGTGGTAGTGCGGAAGTGC |
| MoFpk1(site mutate) UP R | GTTGTTTTTAAGGCAGACATGTTGGGAATTGTGTCCGGT |
| MoFpk1(site mutate) Down F | GGGCAAAGGAATAGAGTAGATGCTGGTCAGTTGGGCTTGG |
| MoFpk1(site mutate) Down R | CGGCCAGTGCCAAGCTTTGTCCGTAAGTTCAGGGT |
| MoFpk1^K230R^ F1 | ATGTCTGCCTTAAAAACAAC |
| MoFpk1^K230R^ R2 | CATTCATTGTTGACCTCCACTACTACTTATCATCTCCATAGT |
| MoFpk1^K230R^ R1 | GTCCTTGTCCAACATCCTGAGGGCAAAGAGCTTATCTC |
| MoFpk1^K230R^ F2 | ATGTTGGACAAGGACGAAATG |
| MoFpk1^D326A^ F1 | ATGTCTGCCTTAAAAACAAC |
| MoFpk1^D326A^ R2 | CATTCATTGTTGACCTCCACTACTACTTATCATCTCCATAGT |
| MoFpk1^D326A^ R1 | GTTCTCAGGCTTGAGAGCTCGGTAGATCAAACCGTTC |
| MoFpk1^D326A^ F2 | CTCAAGCCTGAGAACATCCT |
| MoFpk1^D354A^ F1 | ATGTCTGCCTTAAAAACAAC |
| MoFpk1^D354A^ R2 | CATTCATTGTTGACCTCCACTACTACTTATCATCTCCATAGT |
| MoFpk1^D354A^ R1 | CATGGTGGGCATGCCGGCCTGACCTGAGATCTTTG |
| MoFpk1^D354A^ F2 | GGCATGCCCACCATGATAGTC |
| HPH F | TAGTGGAGGTCAACAATGAATG |
| HPH R | CATCTACTCTATTCCTTTGCCC |
| HPHyz R | GATGCAATAGGTCAGGCTCTC |
| qHPH F | CGATAACTTGGTGCGTTTGTC |
| qHPH R | TTGGATGCTTGGGTAGAATAGG |
| Bar-cx-F | GTCGCTGTCATCGGGCTGCCCAACG |
| Bar-cx-R | AATGTCCTCGTTCCTGTCTG |
| Actin(MGG_03982) F | ACAATGGTTCGGGTATGTGC |
| Actin(MGG_03982) R | CGACAATGGACGGGAAGAC |
| 40S(MGG_02872) F | ACAAGCTCAAGACCCTCGTC |
| 40S(MGG_02872) R | GGTGGTGATGGTGAAGCAG |
| qSUR F | CAAGGAGTGGAAGGAGAAGTG |
| qSUR R | CCGGTTGTGATGTAGGTCATG |
| RT-MSB2 F | CCACCCCTAATACATCCGTTG |
| RT-MSB2 R | TCGGGCTAAGGGTATCAGAG |
| RT-Mst12 F | GACCCTACATCTGCCCTTATTG |
| RT-Mst12 R | AGTACTCTTCCTCGTCCTCG |
| RT-CON7 F | ACCATTTAAACGCGCATGTG |
| RT-CON7 R | CGTTCCTCGTCTGCCTTG |
| RT-SFL1 F | GTTCCTAACCCTGACAACCC |
| RT-SFL1 R | AGCTGTGAGAAGTAATGCCG |
| RT-SHO1 F | GAACACTCAAGAGGACAGTACG |
| RT-SHO1 R | CACAATGGATGCGATGAAAGC |
| RT-CPKA F | TGGGCGAAGTCAAGAATCC |
| RT-CPKA R | TTGCTACTGGGTTTGGGAAG |
| RT-MAE1 F | GCTTTCTGGTCAACTTCATGG |
| RT-MAE1 R | GTGTCTAATGCTGAGTGTGGAG |
| RT-PMK1 F | ATTTCCATCCTCGACATCCAG |
| RT-PMK1 R | GTCTGGTAGATGAAGTACTGGC |
| RT-MPS1 F | CAAGAAAATCCTAGCCAAGCG |
| RT-MPS1 R | GGTCTCGTTGAAGTTATCTGGG |
| RT-OSM1 F | GGAGAAGCAGTTCATCCAGTAC |
| RT-OSM1 R | AAGTCGCAGATCTTCAAGTCG |
| RT-HOX2 F | AAGAACCACAAGCCTACCAG |
| RT-HOX2 R | TCCCTTTTCCTTTGCCCTC |
| RT-COS1 F | CCGGGATATGATTGCCTCTG |
| RT-COS1 R | CCCTTCGCTGTGTACTGTG |
| MGG_13647RT-F | TGGAGAAGGAGTGGGACG |
| MGG_13647RT-R | ACATGCTAACGACACCCAC |
| MGG_00057RT-F | CAGATATGTGAGGTGGGACATC |
| MGG_00057RT-R | TGTTGATGGCGTCGGTG |
| MGG_07890RT-F | GGAACTATTCGGACCCATCTG |
| MGG_07890RT-R | GTGGTCTATCTCCTTTTGGTCG |
| MGG_01925RT-F | GTCTTCGGGAGGCTATTCATG |
| MGG_01925RT-R | TCTCGTCAAACATCCCCTTG |
| MGG_05401RT-F | TCTTGTCCATGAAGTCTGCG |
| MGG_05401RT-R | GATACCCCAGTTCTCTTCGTC |
| MGG_03880RT-F | CCGGAAATCCCTACAGAACAA |
| MGG_03880RT-R | ACACGCCAGAGTACTTGATG |
| MGG_00220RT-F | GAAGTTCCCTCTGGTTGTCG |
| MGG_00220RT-R | CATAGGATTGTGCACCAACAC |
| MGG_10005RT-F | TCAGGCTCTTTTGTAGGCG |
| MGG_10005RT-R | AGCGGGTCGTGTTCATG |
| MGG_00187RT-F | GAAGCATACAAGTACCCGAGG |
| MGG_00187RT-R | ACTCCGATCTCTTTGCCAAC |
| MGG_13626RT-F | AACGCTTCGCCTATGGAG |
| MGG_13626RT-R | TTGAGGCCCATGCTGATG |
| MGG_13793RT-F | CCTGGTACACATTTCCACCTC |
| MGG_13793RT-R | CAGAGATCACATAGCGGGC |
| MGG_08074RT-F | ACGTCTTTGAGGCCAACAG |
| MGG_08074RT-R | CTTTTCGCGGCTCCATTTG |
| MGG_06888RT-F | TGGATTGACAGTGAAGGTGG |
| MGG_06888RT-R | AACGGGCTTGAGGAACAC |
| MGG_13429RT-F | GGATTTCTTTTCACCGTTCGTC |
| MGG_13429RT-R | CTACCTTCAGCAACCCACTC |
| MGG_01281RT-F | GTGGTCTTTGTGACTGCTTTC |
| MGG_01281RT-R | GATCGCCTTGGTCTGGTAG |
| MGG_09234RT-F | ACCTGGCAGAAAAGAACGAG |
| MGG_09234RT-R | CGAGGTCGATGCCAAAGG |
